# Conventional Therapies Deplete Brain-Infiltrating Adaptive Immune Cells in a Mouse Model of Group 3 Medulloblastoma Implicating Myeloid Cells as Favorable Immunotherapy Targets

**DOI:** 10.3389/fimmu.2022.837013

**Published:** 2022-03-03

**Authors:** Zahra Abbas, Courtney George, Mathew Ancliffe, Meegan Howlett, Anya C. Jones, Mani Kuchibhotla, Robert J. Wechsler-Reya, Nicholas G. Gottardo, Raelene Endersby

**Affiliations:** ^1^ Centre for Child Health Research, University of Western Australia, Perth, WA, Australia; ^2^ Brain Tumour Research Program, Telethon Kids Cancer Centre, Telethon Kids Institute, Perth, WA, Australia; ^3^ School of Medical and Health Sciences, Edith Cowan University, Perth, WA, Australia; ^4^ School of Veterinary and Life Sciences, Murdoch University, Perth, WA, Australia; ^5^ Cancer Centre Core Research, Telethon Kids Cancer Centre, Telethon Kids Institute, Perth, WA, Australia; ^6^ NCI-Designated Cancer Center, Sanford Burnham Prebys Medical Discovery Institute, La Jolla, CA, United States; ^7^ Department of Paediatric and Adolescent Oncology and Haematology, Perth Children’s Hospital, Perth, WA, Australia

**Keywords:** medulloblastoma, immune microenvironment, microglia, immunocharacterization, chemotherapy, craniospinal irradiation, group 3

## Abstract

Medulloblastoma is the most common childhood brain cancer. Mainstay treatments of radiation and chemotherapy have not changed in decades and new treatment approaches are crucial for the improvement of clinical outcomes. To date, immunotherapies for medulloblastoma have been unsuccessful, and studies investigating the immune microenvironment of the disease and the impact of current therapies are limited. Preclinical models that recapitulate both the disease and immune environment are essential for understanding immune-tumor interactions and to aid the identification of new and effective immunotherapies. Using an immune-competent mouse model of aggressive *Myc*-driven medulloblastoma, we characterized the brain immune microenvironment and changes induced in response to craniospinal irradiation, or the medulloblastoma chemotherapies cyclophosphamide or gemcitabine. The role of adaptive immunity in disease progression and treatment response was delineated by comparing survival outcomes in wildtype C57Bl/6J and in mice deficient in *Rag1* that lack mature T and B cells. We found medulloblastomas in wildtype and *Rag1*-deficient mice grew equally fast, and that craniospinal irradiation and chemotherapies extended survival equally in wildtype and *Rag1*-deficient mice, suggesting that tumor growth and treatment response is independent of T and B cells. Medulloblastomas were myeloid dominant, and in wildtype mice, craniospinal irradiation and cyclophosphamide depleted T and B cells in the brain. Gemcitabine treatment was found to minimally alter the immune populations in the brain, resulting only in a depletion of neutrophils. Intratumorally, we observed an abundance of Iba1^+^ macrophages, and we show that CD45^high^ cells comprise the majority of immune cells within these medulloblastomas but found that existing markers are insufficient to clearly delineate resident microglia from infiltrating macrophages. Ultimately, brain resident and peripheral macrophages dominate the brain and tumor microenvironment and are not depleted by standard-of-care medulloblastoma therapies. These populations therefore present a favorable target for immunotherapy in combination with front-line treatments.

## Introduction

Medulloblastoma is the most common malignant brain cancer in children, accounting for over 60% of childhood embryonal brain tumors [reviewed in (1)]. Extensive molecular analyses by multiple groups have revealed that medulloblastomas can be classified according to molecular and histopathological features into four major subgroups (WNT, SHH, Group 3, and Group 4) which vary in their clinical outcomes (2, 3). Standard-of-care treatment consists of radiotherapy and chemotherapy following surgical resection which has not changed for decades, survival outcomes have plateaued (4), and severe treatment-induced toxicity remains a major problem for survivors (1). Approximately 30% of children with medulloblastoma will fail conventional therapy (5). While certain molecular features can identify tumors at high risk of treatment failure, including amplification and/or overexpression of *MYC* in Group 3 medulloblastoma (6), limited therapeutic options exist for patients following relapse and there are minimal genetic changes in Group 3 tumors that can be therapeutically targeted at this disease stage (7). Consequently, there is an urgent and unmet need to identify new therapies for the treatment of medulloblastoma and to improve quality-of-life following disease control.

Immunotherapy has arisen as a possible adjunct to conventional therapy to improve the efficacy and mitigate the profound neurotoxicity of current medulloblastoma treatments; however, there have been few studies defining the mechanisms by which medulloblastomas evade anti-tumoral immune activity. Despite the successes immunotherapies have had in other cancers, no clinically approved immunotherapy has had proven success in clinical trials for medulloblastoma to date. Although there are several different immunotherapeutic approaches currently in clinical trials for children with medulloblastoma, including immune checkpoint inhibitors, oncolytic viruses, and dendritic cell vaccines, all of these are early phase studies, and none have progressed beyond phase 2. Moreover, as with many early phase clinical trials for pediatric cancer, these agents are being evaluated in children with recurrent or relapsed disease; whereas past clinical trial experience indicates that new therapies for medulloblastoma have the greatest chance of success when applied early in the course of the disease. This is because relapsed medulloblastoma is typically highly treatment resistant and a patient’s likelihood of responding to salvage therapy is low at this disease stage (<5% long-term survival) (5, 8, 9). It is therefore important that new immuno-therapies being considered for medulloblastoma are rationally designed based on the immune cells present within tumors and tested for efficacy in combination with standard first-line therapies like radiotherapy and chemotherapy. However, studies investigating the impact of radiotherapy and chemotherapy on the immune microenvironment of medulloblastoma are limited. As a result, it is poorly understood how compatible standard therapies are with existing or emerging immunotherapeutics.

Here, we have utilized both immune-competent and immunodeficient murine models of aggressive *Myc*-driven medulloblastoma (10) and characterized adaptive and innate immune cell infiltration in the brain. We describe the impact of the adaptive immune system on tumor growth and treatment response using *Rag1* knockout mice. These mice are deficient in V(D)J recombination, resulting in the arrest of T and B cell differentiation at an early stage and subsequent severe combined immunodeficiency (11). In addition, we have defined how the immune microenvironment changes in response to clinically-relevant fractionated craniospinal irradiation (CSI) protocols, or to the clinically used medulloblastoma chemotherapies cyclophosphamide (CPA) or gemcitabine (GEM). We show that these first-line therapies deplete lymphocyte populations in the brain/medulloblastoma microenvironment, and recommend these impacts be considered when designing future up-front treatment protocols that incorporate immunotherapies for medulloblastoma.

## Materials and Methods

### Preclinical Models

6-12 week old female C57Bl/6J (WT) mice were obtained from the Animal Resource Centre (Perth, Australia). 6-12 week old female C57Bl/6J *Rag1^-/-^* (Rag1KO) mice, that lack mature T and B cells (11), were obtained from an on-site breeding colony at the Telethon Kids Institute Bioresources Facility. Mice were group-housed in a pathogen-free facility at the Telethon Kids Institute (12:12 hour light:dark cycle) with access to standard chow and water *ad libitum*. Mice received sunflower seeds during treatment for enrichment and to maintain healthy weight. All animal procedures were approved by the Animal Ethics Committee of the Telethon Kids Institute and performed in accordance with Australia’s Code for the Care and use of Animals for Scientific Purposes.

The murine allograft model of aggressive *Myc*-amplified Group 3 medulloblastoma (Myc/p53^DD^) was generated through retroviral-driven expression of *Myc* ^T58A^, a dominant negative carboxy-terminal fragment of *Tp53*, GFP and firefly luciferase in CD133-positive cerebellar stem cells as previously described (10). For intracranial implantation, Myc/p53^DD^ cells were harvested from female C57Bl/6J donor mice, suspended in Matrigel (BD Biosciences), and 5,000 cells were implanted per mouse as previously described (12).

Tumor size was monitored using bioluminescence imaging with an IVIS Spectrum (Caliper, USA). Prior to imaging, fur was removed with electric clippers and depilatory cream. Mice received intraperitoneal injections of D-Luciferin [15 mg/kg in Dulbecco’s phosphate-buffered saline (DPBS)] and were anesthetized with isoflurane. During image acquisition, isoflurane was maintained at 1.5-1.8% in oxygen (flow rate 0.5 L/min) and images were acquired every minute for 10 minutes until peak photon flux was recorded. Bioluminescence was used as a surrogate measure of tumor burden and mice were randomized into groups such that the average flux ± standard deviation (SD) was equal across all groups at the start of treatment.

### Craniospinal Irradiation (CSI)

**Figure 1:**
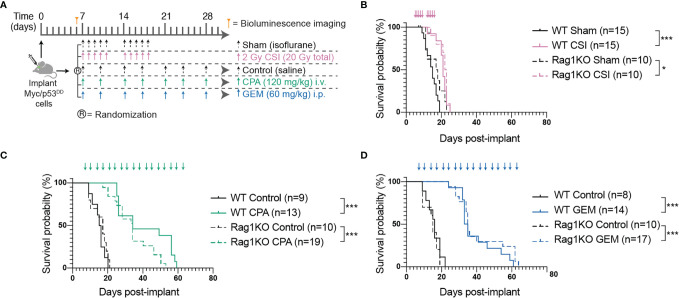
The adaptive immune system does not play a role in Myc/p53^DD^ tumor progression nor treatment efficacy. **(A)** Schematic diagram illustrating the treatment protocols for craniospinal irradiation (CSI), cyclophosphamide (CPA) and gemcitabine (GEM). **(B–D)** Survival curves of multiple independent experiments (minimum of two independent experiments per treatment) of WT (*solid lines*) or Rag1KO (*dashed lines*) mice treated with **(B)** CSI (*pink*), **(C)** CPA (*green*), or **(D)** GEM (*blue*). Arrows on graphs indicate when treatment was administered. The number of mice per group (n) is shown and significant differences between survival curves determined using log-rank tests is indicated (*P < 0.05; ***P < 0.001).

Irradiation was performed using a X-RAD SmART small animal image-guided radiation therapy system (Precision X-Ray, USA) employing cone-beam CT guidance with fully assessed spatial and dosimetric accuracy (13). Treatment planning and dose calculations were performed using Monte Carlo simulations in SmART-Plan software (14). Mice were anesthetized with isoflurane, maintained at 1-2% in air delivered *via* nose cone during treatment. Mice were secured to the irradiation stage with non-adhesive athletic tape to flatten the spine and avoid irradiating abdominal organs. CSI was achieved using two sets of two lateral coplanar beams with 40 mm square collimation delivered to two separate isocenters, with the first set of beams targeting the brain and cervical spine, the second targeting the thoracic and lumbar spine. Mice received a total of 20 Gy CSI fractionated as 10 doses of 2 Gy, delivered on a 5-days-on, 2-days-off schedule for two weeks (15) (**Figure 1A**). For the sham control group, mice were anesthetized with isoflurane on a 5-days-on, 2-days-off schedule for two weeks for an equal length of time per day as the CSI treatment protocol. Animals were humanely euthanized upon the onset of tumor-related morbidity.

### Chemotherapy

Cyclophosphamide (CPA, Baxter) and gemcitabine (GEM, MedChemExpress) were diluted in phosphate-buffered saline (PBS) and delivered twice weekly (day 7, 10, 14, 17, etc.). CPA was delivered intraperitoneally (i.p.) at 120 mg/kg and GEM was delivered intravenously (i.v.) at 60 mg/kg. Control mice received saline injections *via* the equivalent route on the same schedule (**Figure 1A**). Treatment was continued until mice required euthanasia due to tumor-related morbidity.

### Flow Cytometry

Single cell suspensions were prepared from whole brains for flow cytometric analysis. Whole brains were minced on a sterile petri dish with a scalpel blade, prior to addition of 5 mL digestion buffer (100 U/mL Collagenase IV (Life Technologies), 10 U/mL DNAse (Sigma-Aldrich) in Hank’s balanced salt solution (HBSS, Gibco)), followed by trituration to obtain a uniform suspension. The tissue suspension was transferred to a gentleMACS C tube (Miltenyi) and further digested on a gentleMACS Octo Dissociator (Miltenyi) for 30 minutes, with constant stirring at 50 RPM at 37°C. Digestion was halted with the addition of 10 mL cold FACS buffer (2% fetal calf serum, 5 mM EDTA in HBSS) and the suspension was strained through a 100 μm filter (Miltenyi). Red blood cells were lysed with red blood cell lysis solution (Miltenyi), cells were resuspended in 10 mL FACS buffer and strained through a 30 μm filter (Miltenyi). To remove myelin, cells were resuspended in 10 mL of 30% Percoll (Sigma-Aldrich) diluted in FACS buffer and centrifuged at 800 g for 30 min at room temperature. The myelin layer was removed, and cells resuspended in DPBS before staining.

Given that chemotherapy is known to have systemic effects, spleen tissue was routinely collected and analyzed alongside brain tissue as a control to characterize the effects of chemotherapy on immune cells outside the central nervous system. In addition, the spleens of mice treated with CSI would have received some off-target irradiation when radiotherapy was delivered to the thoracic and lumbar spine. Spleen dissociation protocols and results can be found in the **Supplementary Data** (**Supplementary Figure 1**).

**Figure 2:**
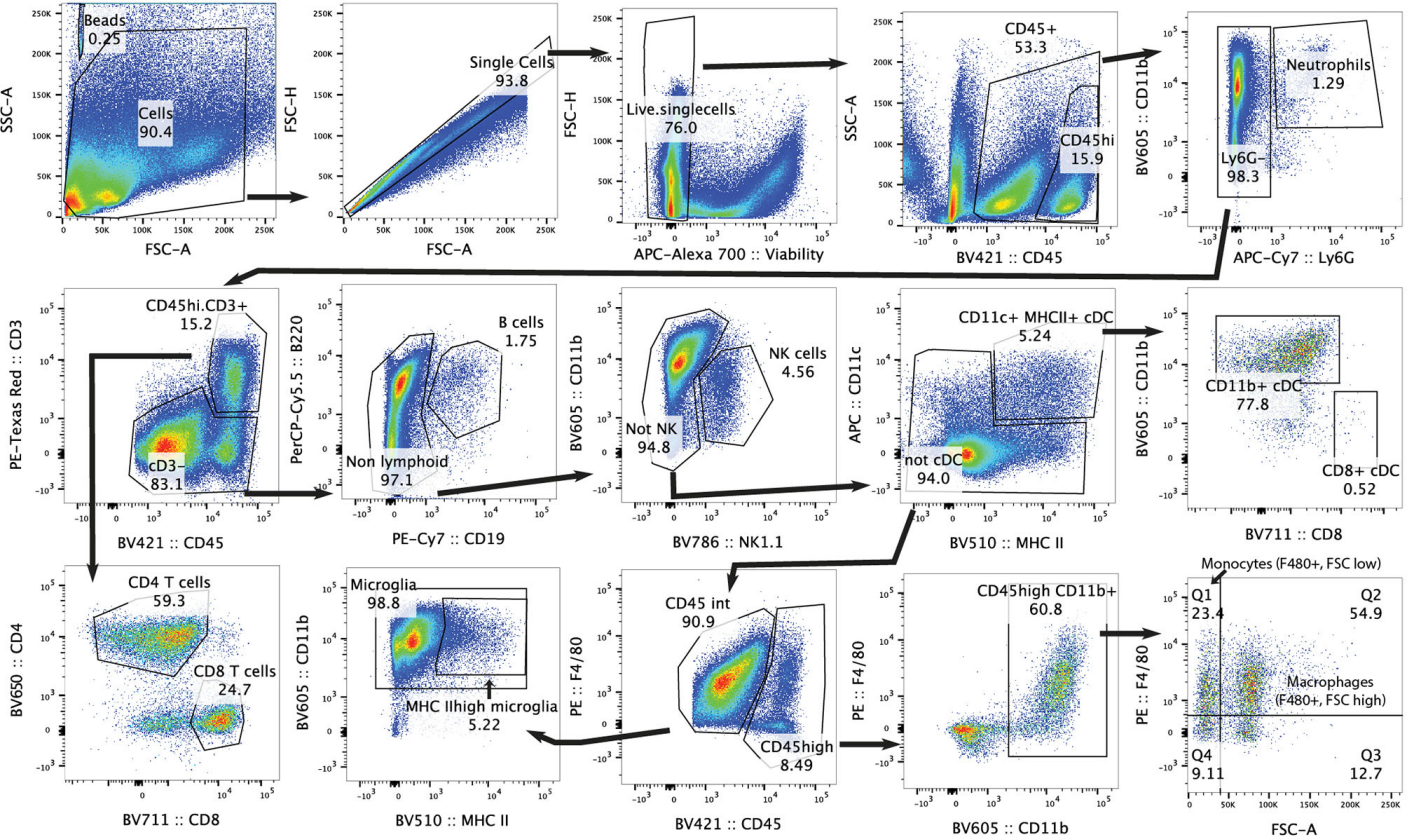
Gating strategy for flow cytometry analysis of mouse brain. Representative plots of gating strategy for immune populations in C57Bl/6J WT brain.

Single cell suspensions were labelled with a viability stain (BD Bioscience Cat #564997) then stained with the following fluorochrome conjugated cell surface marker antibodies: CD45-BV421 (BD Bioscience Cat #563890), IAIE-BV510 (Biolegend Cat #107635), CD11b-BV605 (Biolegend Cat #101257), CD4-BV650 (BD Bioscience Cat #563747), CD8a-BV711 (BD Bioscience Cat #563046), NK1.1-BV786 (Biolegend Cat #108749), B220-PerCP-Cy5.5 (BD Bioscience Cat #552771), F4/80-PE (BD Bioscience Cat #565410), CD3e-PE-CF594 (BD Bioscience Cat #562286), CD19-PECy7 (BD Bioscience Cat #552854), CD11c-APC (BD Bioscience Cat #550261), Ly6G-APC-Cy7 (BD Bioscience Cat #560600). Antibody dilutions and the staining protocol can be found in the **Supplementary Material** (**Supplementary Methods** and **Supplementary Table 1**). Data were acquired on a LSRFortessa X-20 (BD Bioscience, USA) and immune populations were gated using FlowJo (**Figure 2**). The combination of markers used to define different immune populations is described in **Supplementary Table 2**. Positively stained cells are presented as a proportion of all CD45-positive cells. Alternatively, calibration beads (BD Bioscience Cat #556296) were added to cell suspensions to quantify the total numbers of immune cells within each tissue sample by comparing the ratio of bead events to cell events. Population statistics were compared and graphed in GraphPad PRISM v8. Gating strategy for populations from Rag1KO mice are shown in **Supplementary Figure 2**.

For CSI treated mice, immune cell populations were assessed at two experimental time points. Firstly, tissue was harvested 24 hours following the final (10^th^) dose of CSI (referred to as “acute”) to capture transient changes in immune populations following CSI. A second time point was captured approximately 1-2 weeks after the cessation of CSI (referred to as “late stage”), when tumor burden was high and mice were moribund, to determine lasting effects of CSI. For all chemotherapy experiments, brains were analyzed when tumor burden was high and caused morbidity requiring euthanasia. Due to the continual dosing schedule (**Figure 1**), tumor related morbidity and tissue analyses occurred within 2-3 days of chemotherapy dosing. Healthy, non-tumor bearing mice were time-matched to Myc/p53^DD^ bearing mice. To determine proportions of CD45^high^ and CD45^intermediate^ (CD45^int^) tumor-infiltrating immune cells, untreated Myc/p53^DD^ medulloblastomas from C57Bl/6J WT mice were dissected out from the brain, dissociated as above, and labelled with viability stain (BD Bioscience Cat #564997) and CD45-BV421 (BD Bioscience Cat #563890).

### Immunohistochemistry (IHC)

Mice were transcardially perfused with PBS, followed by 4% paraformaldehyde (PFA) in PBS. Brains were further fixed overnight in 4% PFA at 4°C before embedding into paraffin. IHC was performed on 5 μm sections. Briefly, sections were deparaffinized and rehydrated using an ST5010 AutoStainer XL (Leica, Germany). Antigen retrieval was performed using a sodium citrate buffer (1.8 mM citric acid, 8.2 mM sodium citrate), sections were incubated in 3% H_2_O_2_ to block endogenous peroxidases, blocked with 10% normal goat serum in Tris buffered saline containing 0.01% Tween 20 (TBS-T) for one hour at room temperature, and incubated with primary antibodies overnight at 4°C in 2% goat serum in TBS-T. Slides were incubated with biotinylated secondary antibodies, then incubated with an streptavidin-conjugated peroxidase reagent (Elite ABC, Vector Labs). Slides were incubated with NovaRED peroxidase substrate (Vector Labs), counterstained with Gill’s Hematoxylin (Vector Labs), dehydrated, and coverslipped with Permount (Fisher Scientific). Slides were stained with the following antibodies: Iba1 (1:800, Wako Chemicals, Cat #019-19741), Tmem119 (1:300, Abcam, Cat #ab209064). Positively-stained cells with evident nuclei were counted from four 1mm^2^ areas per mouse corresponding to three different areas: normal cortex, areas where the image consisted of 50% tumor and 50% normal brain, or tumor. Data are presented as cells per mm^2^.

### RNA Isolation and Bulk RNA Sequencing

Tumor tissue was dissected from the brain, snap frozen using dry ice and stored at -80°C. Total RNA was isolated from 5-20 mg tumor tissue using the RNeasy Plus Mini Kit (Qiagen) as per the supplied protocol. RNA concentration and purity was assessed on a spectrophotometer (NanoDrop) and total RNA was submitted to GenomicsWA (Perth, Australia) (CSI and Sham samples) or the Australian Genome Research Facility (AGRF) (CPA, GEM, control samples). Samples had an average ± SD RNA integrity number (RIN) of 9.8 ± 0.36 prior to library preparation. Total RNA library preparation (SureSelect, Agilent), rRNA depletion (Ribo-Zero Plus, Illumina) and sequencing were carried out by GenomicsWA or AGRF. Libraries were sequenced on NovaSeq 6000 S1 flow cells as paired-end 150bp reads (Illumina). Raw sequencing data for two independent datasets are available from the European Genome Archives (EGAS00001005847 and EGAS00001005846).

### Pre-Processing, Quality Control (QC) and Exploratory Data Analysis

Adapter and quality trimming were applied using CutAdapt (16). Pre-alignment and post-alignment QC were carried out with FastQC (17) and SAMStat (18) respectively. Reads were aligned to the mouse reference genome (GRCm38) using HISAT2 (19) and quantified at the gene-level with *summarizedOverlaps()* (20). The proportion of mapped reads was 84% (79.7-87%) in CPA/GEM/Control samples and 90% (89.3-91.5%) in CSI/Sham samples.

Data analysis was carried out in the statistical computing environment R (version 4.1.1). Genes with an official MGI Gene Nomenclature Committee symbol and a count per million corresponding to 10 in ≥3 were retained for downstream analysis. Exploratory data analysis was carried out using EDASeq (21) and standard QC plots were used to identify potential outlying samples pre- and post-global-scale median normalization of gene counts. Unwanted variation was removed employing RUVSeq (21).

### Estimation of the Cellular Composition With CIBERSORTx

CIBERSORTx (22) was used to estimate the immune cell proportions in medulloblastoma tissue. A published C57Bl/6J WT whole brain single cell dataset (23) (GEO accession GSE128855) was used as the reference dataset containing 8 annotated brain immune cell types (microglia, B cells, NK/NKT cells, T cells, cDC, monocytes, border associated macrophages, neutrophils). The following parameters were used: disabled batch correction, relative run mode, 100 permutations. Cell fractions calculated by CIBERSORTx in treatment groups were compared to their control (CSI vs Sham, CPA and GEM vs Control) by unpaired two-tailed *t tests*.

### Differential Expression Analysis

Differentially expressed genes were identified using edgeR (24). A linear binomial model was fit to the data and a false discovery rate (FDR) for multiple testing was applied. An adjusted P < 0.05 and an absolute log2 fold change > 0.5 (fold change = 1.5) was deemed significant.

### Pathway Analysis

Up- and downregulated genes were assessed separately for pathways enrichment using InnateDB (25) version 5.4. Enrichment testing is based on a hypergeometric distribution and P values are corrected using the Benjamini-Hochberg method for multiple testing.

### Upstream Driver Analysis

Putative molecular drivers of the observed gene expression patterns were identified using upstream regulator analysis from Ingenuity Systems KnowledgeBase (26). Significance testing is based on a Fisher’s exact test, testing for enrichment against known upstream drivers. The Benjamini-Hochberg method was used to correct for multiple testing. An adjusted P-value < 0.01 and absolute z-score > 2.0 (predicting activation/inhibition of the driver) were deemed significant.

### Statistical Analyses

Kaplan-Meier survival curves were compared using the log-rank test. In each survival experiment, treatment groups were compared to their equivalent controls. Unpaired two-tailed *t* tests were used to compare immune populations for flow cytometry data. Treatments were compared to their control (CSI vs Sham, CPA and GEM vs Control). For CSI experiments, each time-point group was only compared to its time-matched sham (late-stage/acute). Being an exploratory study, P values are stated without multiplicity adjustments (27) and significant differences were defined as P < 0.05 in flow cytometry, IHC, and CIBERSORTx comparisons.

## Results

### Myc/p53^DD^ Tumor Growth Is Unaffected by a Functional Adaptive Immune System

The majority of preclinical medulloblastoma mouse models utilize immune-deficient strains and the role of the immune system in medulloblastoma growth is poorly defined. To address this, we took advantage of a murine model of Group 3 medulloblastoma that engrafts in C57Bl/6J mice following intracranial implantation and compared tumor growth in wildtype C57Bl/6J mice and C57Bl/6J mice deficient in *Rag1*. Tumor-free survival of control mice (sham/isoflurane or control/saline) was not different between WT and Rag1KO animals, indicating that growth of this model of Group 3 medulloblastoma is not impacted by the presence or absence of mature T and B cells (**Figure 1**). To determine the role of the adaptive immune system in response to conventional first-line therapies, medulloblastoma-bearing mice of either strain were treated with CSI or two DNA-damaging chemotherapies used as part of clinical care: CPA or GEM (28) (NCT01878617) (**Figure 1A**). Compared to control groups, CSI prolonged median survival similarly in WT mice (21 compared to 15 days; P<0.0001) and Rag1KO mice (22.5 compared to 17.5 days; P=0.03). Likewise, response of Myc/p53^DD^ tumors to chemotherapy was similar in WT and Rag1KO mice. CPA prolonged median survival to 34 days in both WT mice (P<0.001) and Rag1KO mice (P<0.0001); while GEM prolonged median survival from 16 days to 34 days in WT mice (P<0.0001) and from 15 to 35 days in Rag1KO mice (P<0.0001). These results indicate that treatment-mediated tumor control is independent of T and B cells in this model of medulloblastoma (**Figures 1B–D**).

### Group 3 Medulloblastoma Growth Stimulates Immune Cell Influx Into the Brain

The finding that the adaptive immune system did not appear to modulate response to therapy was surprising given previous reports of increased CD8^+^ T cells in murine Group 3 medulloblastoma (29). Furthermore, it is unknown what impact conventional medulloblastoma treatments have on immune cells within the brain. To understand whether these treatments were altering the immune populations in the whole brain, mice harboring Myc/p53^DD^ tumors were administered treatment (or control) as described above, and the immune cells present in brain tissue were assessed by flow cytometry when mice required euthanasia due to tumor-related morbidity, using the gating strategy defined in **Figure 2**.

**Figure 3:**
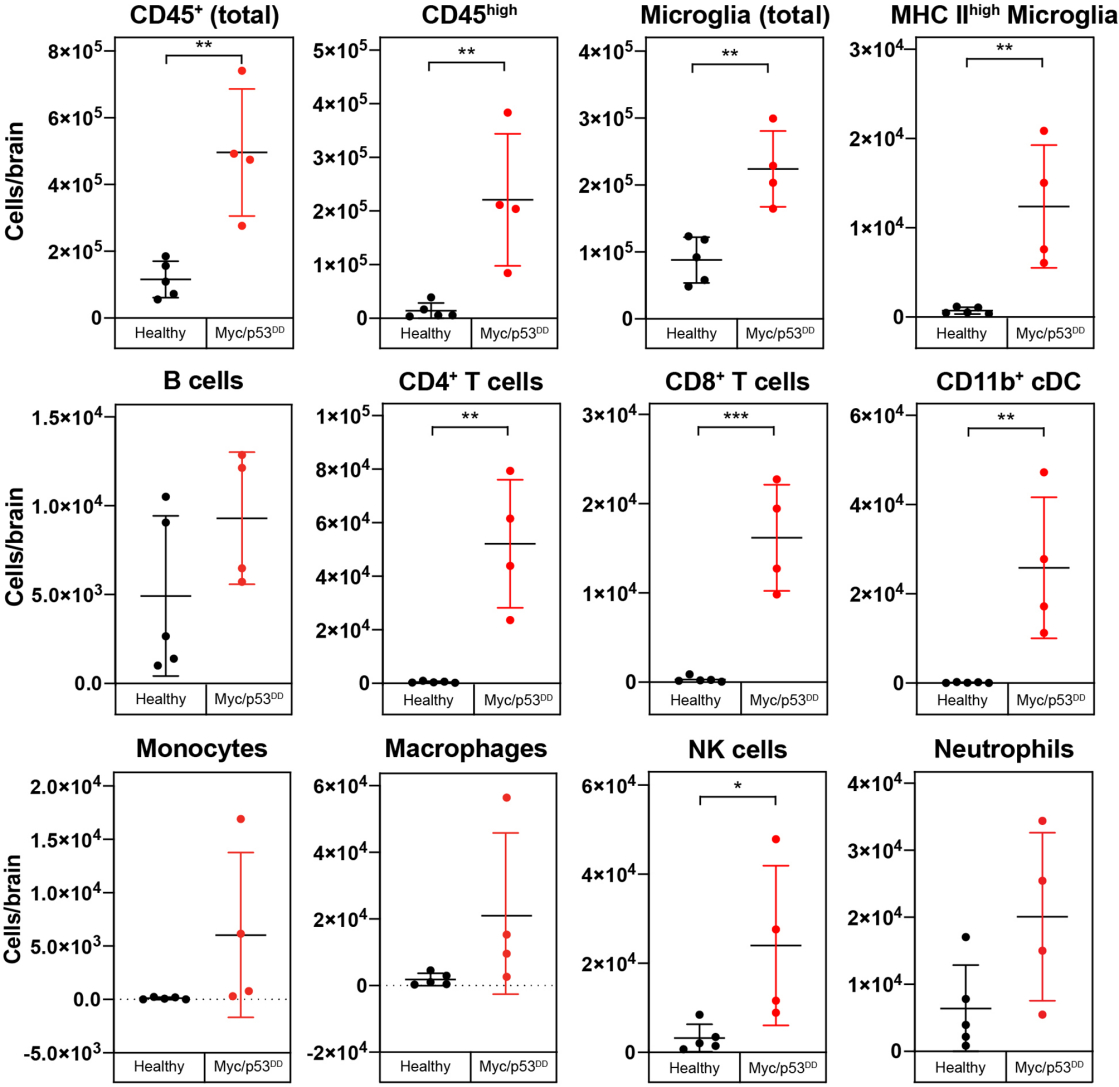
Growth of Myc/p53^DD^ medulloblastoma increases the numbers of infiltrating and resident immune cells in the brain. The immune cell populations from healthy brains of C57Bl/6J WT mice (*black circles*, n=5) were determined using flow cytometry and compared with the brains of WT mice bearing Myc/p53^DD^ tumors (*red circles*, n=4). Beads were used and the entire sample was analyzed to determine the total number of the indicated cells in each sample. Tumor-containing brains had significantly higher overall counts of immune cells (CD45^+^), both infiltrating (CD45^high^) and resident (microglia) compared to healthy brain. Moreover, an increase in activated microglia (MHC II^high^), CD4^+^ T cells, CD8^+^ T cells, NK cells, and CD11b^+^ cDCs was observed in Myc/p53^DD^ tumor bearing brains. No significant change in neutrophils, B cells, monocytes or macrophages was observed. Horizontal lines indicate the mean and error bars indicate SD. Comparisons shown to be statistically significant by *t* test are shown (*P < 0.05; **P < 0.01; ***P < 0.001).

In the absence of treatment, we observed that medulloblastoma growth induced an overall influx of immune cells into the brain (**Figure 3**). Brain-resident microglia in adult mice express lower levels of CD45 compared to bone-marrow derived immune cells, thus can be distinguished using flow cytometry (30, 31). The addition of calibration beads enabled us to analyze absolute cell numbers in the cell suspensions of the entire brain and we observed a significant increase in immune cell numbers (CD45^+^), both CD45^high^ and microglia (CD45^int^ CD11b^+^) in the brains of tumor-bearing WT mice compared to healthy age-matched brains. Moreover, we observed increased counts of activated microglia (CD45^int^ CD11b^+^ MHC II^high^) (32), classical dendritic cells (cDCs) (CD45^high^ CD11c^+^ MHC II^+^, CD11b^+^), NK cells (CD45^high^ NK1.1^+^), CD4^+^ T cells (CD45^high^ CD3^+^ CD4^+^), and CD8^+^ T cells (CD45^high^ CD3^+^ CD8^+^), (**Figure 3**). CD8^+^ cDCs accounted for a very small proportion of the immune cells, and their counts are not shown. No significant changes were detected in neutrophil (CD45^high^ Ly6G^+^), B cell (CD45^high^ CD19^+^ B220^+^), monocyte (CD45^high^ CD11b^+^ F4/80^+^ FSC^low^) or macrophage (CD45^high^ CD11b^+^ F4/80^+^ FSC^high^) numbers between normal brains or brains harboring Myc/p53^DD^ medulloblastomas.

To further demonstrate the changes in immune cell populations caused by medulloblastoma growth in WT mouse brain we compared the changes in immune cell populations relative to each other by quantifying differences as a percentage of all CD45^+^ cells. As a proportion of all CD45^+^ immune cells in the brain, significant increases in cDCs, CD4^+^ T cells, CD8^+^ T cells, and activated microglia were observed in tumor-bearing brains compared to healthy brains. As a consequence of this influx, the proportion of microglia relative to all immune cells was decreased in tumor-bearing brains compared to healthy brain (**Supplementary Figure 3**), although as described above, microglial numbers were increased overall.

In Rag1KO mice, growth of Myc/p53^DD^ medulloblastoma also induced an increase in the absolute numbers of microglia in the brain as well as an influx of bone marrow-derived immune cells in the absence of treatment. In addition, we observed a significant increase in CD11b^+^ cDCs, NK cells, neutrophils, and monocytes (**Supplementary Figure 4**), reiterating the finding that even in mice lacking an adaptive immune system, medulloblastoma growth induces influx of bone marrow-derived immune cells to the brain. As observed in WT mice, Myc/p53^DD^ tumor growth significantly elevated the number of activated microglia in Rag1KO brains.

### CSI Transiently Depletes Bone Marrow-Derived Immune Populations in the Brain

**Figure 4:**
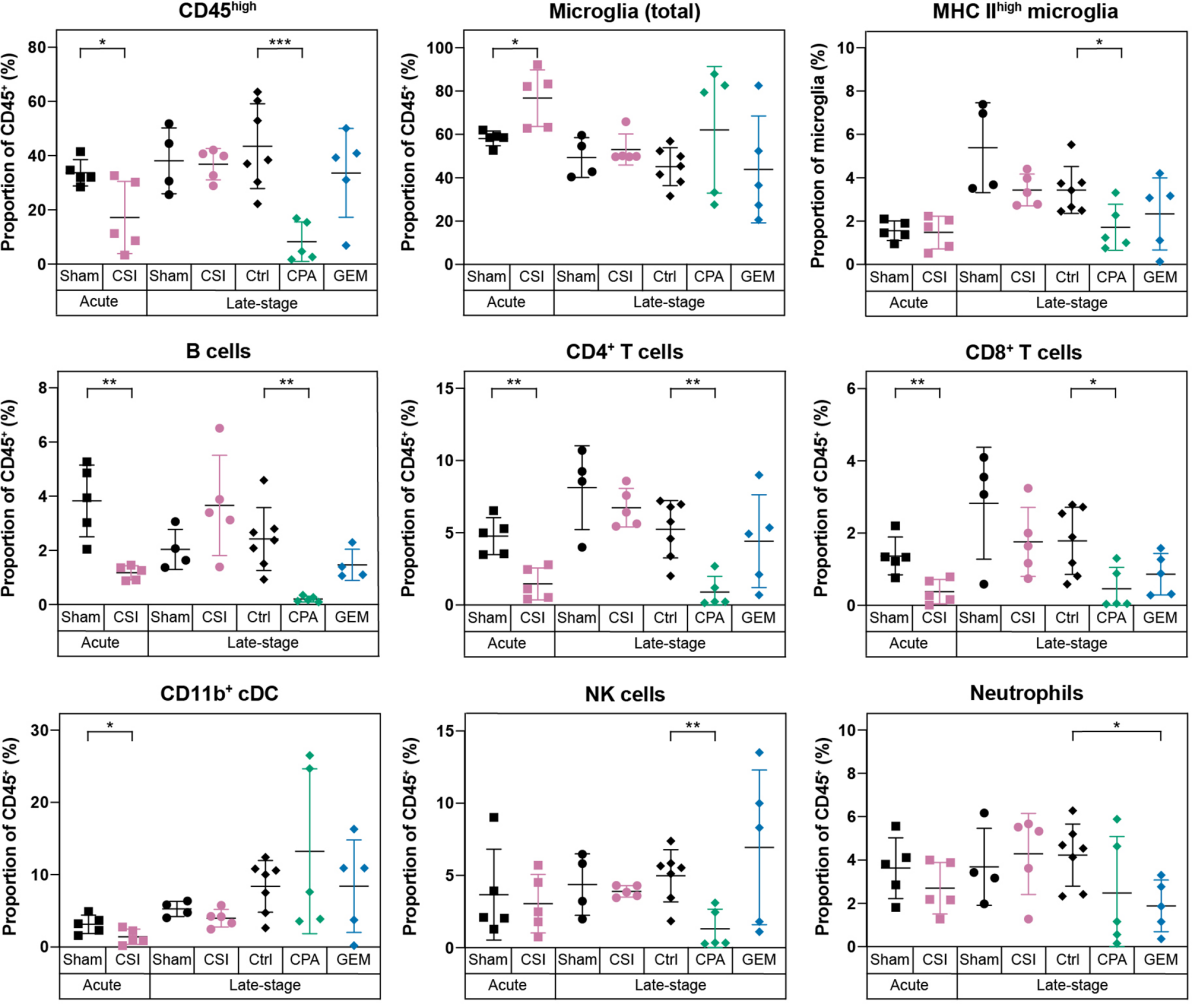
Radiotherapy and chemotherapy alter the immune microenvironment in the brains of C57Bl/6J WT mice with Myc/p53^DD^ medulloblastoma. Immune cell populations (shown as a percentage of all CD45^+^ cells) in WT brains harboring medulloblastoma following a range of different treatments are shown. From left to right, mice with Myc/p53^DD^ medulloblastoma were treated with either sham (*black squares*, n=5) or CSI (*pink squares*, n=5) and harvested 24 hours after the tenth dose (labeled “Acute”), sham (*black circles*, n=4) or CSI (*pink circles*, n=5) and harvested upon the development of tumor-related morbidity (labeled “Late stage”), or after treatment with saline (“Ctrl”, *black diamonds*, n=7), CPA (*green diamonds*, n=5), or GEM (*blue diamonds*, n=5) and harvested upon the development of tumor-related morbidity (also labeled “Late stage”). Fractionated CSI resulted in a temporary depletion of infiltrating CD45^high^ immune cells. Specifically, B cells, CD4^+^ T cells, CD8^+^ T cells, and CD11b^+^ cDCs were decreased (*pink squares*) and in consequence a proportional increase of microglia was observed, whereas this was not observed at a later time point (*pink circles*). CPA reduced the abundance of B cells, CD4^+^ T cells, CD8^+^ T cells, and NK cells, while GEM only significantly depleted neutrophils. Horizontal lines indicate the mean and error bars indicate SD. Each treatment group was compared to the appropriate treatment and time-point matched control by *t* test, with statistically significant differences indicated (*P < 0.05; **P < 0.01; ***P < 0.001).

Given this understanding of how the immune microenvironment of brain is altered with medulloblastoma growth, we next characterized the effects of clinical treatments on the immunology of medulloblastoma, starting with CSI. We analyzed brains at two time points following CSI – 24 hours after the final dose of radiation (acute), and at high tumor burden when mice required euthanasia (late-stage) – to determine transient and long-term impacts. In WT mice, CSI resulted in a significant, but transient, reduction in infiltrating bone marrow derived immune cells (CD45^high^). The proportion of CD4^+^ T cells, CD8^+^ T cells, B cells, and CD11b^+^ cDCs in the brain were all observed to be significantly reduced 24 hours following delivery of the last CSI fraction (**Figure 4**, *squares*). In consequence, the proportion of microglia as a percentage of the total immune milieu was significantly higher in CSI treated mice compared to sham treated mice at this time point (**Figure 4**, *squares*). Proportions of monocytes and macrophages were unchanged with treatment (**Supplementary Figure 5**). In contrast, when we examined acute CSI-induced changes in the brains of Rag1KO mice with Myc/p53^DD^ medulloblastoma, only a significant reduction of NK cells was observed 24 hours following CSI (**Supplementary Figure 6**, *squares*). WT mouse brains were also assessed upon the development of tumor-related morbidity, which occurred 1-2 weeks following the cessation of CSI. No differences in immune cell populations were detected in CSI treated mice compared to time-matched controls at this late stage (**Figure 4**, *circles*), indicating that immune population changes induced by CSI in the brain were temporary. These findings contrast recent reports of irradiation-mediated enhanced immune cell infiltration in a model of SHH medulloblastoma (33).

### CPA Depletes Infiltrating CD45^high^ Immune Cells While GEM has Minimal Impacts on the Immune Microenvironment

Chemotherapeutics, including first-line medulloblastoma drugs CPA and GEM, can achieve an anti-tumor response directly *via* DNA damaging activity, or indirectly *via* induction of an immune response through immunogenic cell death, stimulating immune effectors, or inhibiting immune suppressors [reviewed in (34)]. Thus, we sought to characterize the effects of these clinically used chemotherapies on the immune cells within medulloblastoma bearing brains. Brains were analyzed when tumor burden was high (“Late-stage”), which was within 3-4 days of the last chemotherapy dose due to the continual dosing protocol. WT mice treated with CPA had significantly lower proportions of infiltrating immune cells in the brain compared to controls. Proportions of CD4^+^ T cells, CD8^+^ T cells, B cells, NK cells and activated microglia were all significantly reduced in CPA treated mice (**Figure 4**, *green diamonds*). It is known that CPA can cause leukopenia [reviewed in (35)]. Consistent with this, significant decreases in multiple immune cell populations were also observed in the spleens of mice treated with CPA (**Supplementary Figure 1**). In contrast, GEM treatment had minimal effects on the immunology of WT mouse brain, resulting only in a proportional reduction of neutrophils (**Figure 4**, *blue diamonds*).

Surprisingly, chemotherapy-induced immunodepletion was not observed in the brains of Rag1KO mice. Instead, we observed a significant increase in CD45^high^ immune cells in Rag1KO mice treated with either CPA or GEM (**Supplementary Figure 6**). The increase of CD45^high^ cells was higher in CPA treated mice, and we observed a concomitant decrease in the proportion of microglia in CPA treated Rag1KO brains.

### Medulloblastoma-Infiltrating Myeloid Cells Express Iba1 but Not the Microglial Marker Tmem119

**Figure 5:**
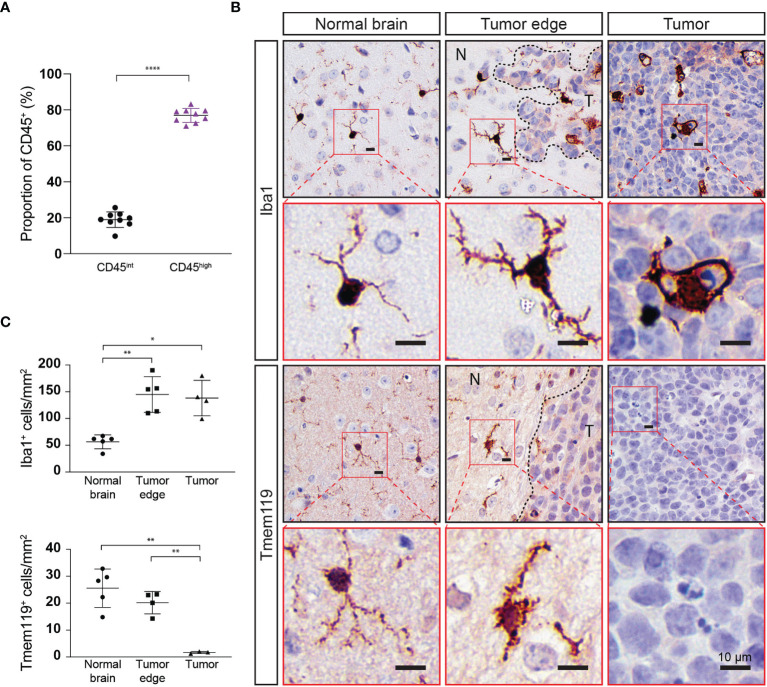
Immune cell populations within Myc/p53^DD^ tumors in WT mouse brain highly express CD45 and do not express Tmem119. **(A)** Myc/p53^DD^ medulloblastomas (n=8) were dissected away from normal C57Bl6/J WT brain and cells were analyzed by flow cytometry for CD45^int^ (*black circles*) or CD45^high^ immune cells (*purple triangles*). Horizontal lines indicate the mean and error bars indicate SD. Comparison by paired *t test* is shown (****P < 0.0001) demonstrating that CD45^high^ immune cells account for the majority of intratumoral immune cells within Myc/p53^DD^ tumors. **(B)** WT mouse brain implanted with Myc/p53^DD^ medulloblastoma were examined using IHC for Iba1 and Tmem119. Representative low (*black border*) and high (*red border*) magnification images of Iba1 (*top*) or Tmem119 (*bottom*) expressing cells demonstrate a resting or ramified appearance in the normal brain (*left*), but have an activated or ameboid appearance around the edge of medulloblastomas (*middle*, *N* indicates normal brain and *T* indicates tumor), characterized by an increase and thickening of membrane projections. Within tumors (*right*), Iba1^+^ cells appear phagocytic, while Tmem119 staining is absent. Nuclei have been counterstained with hematoxylin and the scale bar on each image indicates 10 µm. **(C)** Quantitation of Iba1 or Tmem119 expressing cells from the brain regions indicated. Each symbol represents an individual mouse, horizontal lines indicate the mean and error bars indicate SD. Each area was compared using t test, with statistically significant differences indicated (*P < 0.05; **P < 0.01).

Not surprisingly, our results show that microglia dominate the brain/medulloblastoma immune microenvironment; however, our flow cytometry data did not indicate the spatial distribution of these cells or define if they were interacting with medulloblastoma cells, or instead if they were retained in normal brain. To determine intratumoral distribution of resident (CD45^int^) and infiltrating (CD45^high^) immune cells, we dissected out Myc/p53^DD^ tumors from WT mice and assessed proportions of intratumor immune cells with flow cytometry. We found that immune cells account for only 1-2% of the cells within these tumors, and that a majority (76.9 ± 3.88%) of the immune cells within the tumor were CD45^high^ (**Figure 5A**). However, it has been shown that microglia may up-regulate CD45 under pathological conditions; therefore, our approach of delineating microglia from tumor-infiltrating macrophages on the basis of intermediate versus high CD45 expression may be insufficient (36, 37). To characterize and further delineate the location of resident microglia and infiltrating peripheral myeloid cells in the brain and within Myc/p53^DD^ medulloblastomas, we performed IHC for two myeloid cell markers, Iba1 and Tmem119. Cells staining positively for Iba1, which is a marker of both bone marrow derived macrophages and microglia (38), were observed throughout the brain parenchyma and tumors. The morphology of cells stained with Iba1 varied from ramified in the normal brain, which typifies the resting state of microglia, to a more ameboid state, which typifies the active state, for cells located at the tumor periphery and within medulloblastomas (**Figures 5B, C**). Cells staining positively for the marker Tmem119, reported to be a specific marker of microglia (39), were observed throughout the normal brain displaying ramified morphology. Tmem119 also stained cells around the tumor edge and these cells displayed more ameboid morphology. However, IHC staining for Tmem119 was completely absent within the tumors suggesting these cells were not microglia (**Figures 5B, C**), although this is inconsistent with our flow cytometry findings which indicated that at least 23% of intratumoral immune cells should be microglia based on their lower expression of CD45 (CD45^int^, **Figure 5A**). Together, our findings suggest that either bone-marrow derived immune cells comprise the majority of the intratumoral immune cells and that microglia do not penetrate the tumor, or, given reports of microglia upregulating CD45 in pathological conditions (36, 37), that microglia downregulate Tmem119 and upregulate CD45 in response to medulloblastoma.

### Immunological Signatures in Medulloblastoma Are Poorly Interpretable Using Bulk RNA Sequencing

**Figure 6:**
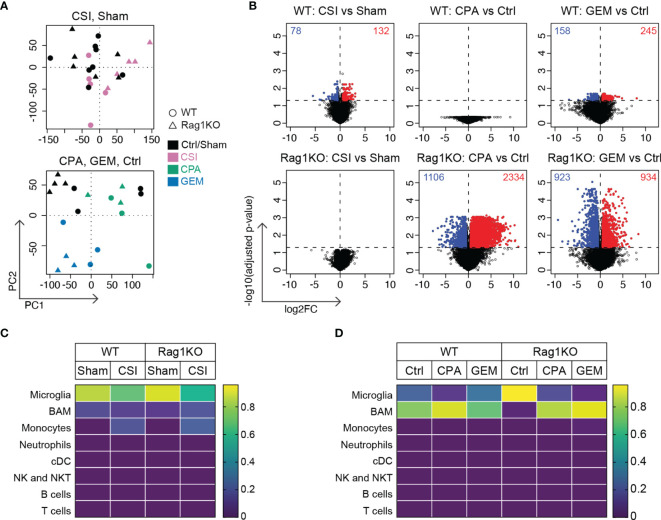
Radiotherapy and chemotherapy induce distinctly different gene expression changes in Group 3 medulloblastoma in immune-competent versus immune-deficient mice. RNA sequencing was performed on Myc/p53^DD^ medulloblastomas from either C57Bl/6J WT mice (*circles*) or C57Bl/6J Rag1KO mice (*triangles*) that were harvested upon the development of tumor-related morbidity following treatment with sham, CSI, saline, CPA, or GEM. **(A)** Principal component analysis plots showing sham treated tumors (*black*) compared with CSI treated tumors (*pink*, upper panel), or saline treated tumors (Ctrl, *black*) compared with CPA (*green*) or GEM (*blue*) treated tumors (lower panel). **(B)** Volcano plots showing DEGs identified using EdgeR in response to CSI, CPA, and GEM treatment in tumors from WT or Rag1KO mice compared to their respective controls (*red* = upregulated*, blue* = downregulated) for the six comparisons indicated. The number of significantly differentially expressed genes is shown in each plot. **(C)** Immune cell fractions in WT and **(D)** Rag1KO tumor tissue were estimated using CIBERSORTx. Color scale indicates the predicted fraction of the indicated immune cells deconvoluted from bulk tumor transcriptome data. CSI did not significantly alter predicted immune fractions in either strain, nor did chemotherapy in WT tumors. Despite non-significant deconvolution, a reduction in microglial signatures was observed in Rag1KO mice following treatment with either CPA (P=2.0x10^-4^) or GEM (P=2.0x10^-5^) compared to control, with an increase in transcripts associated with border associated macrophages (BAMs) (P=0.04 and P=1.8x10^-5^ respectively). Number of mice in each group were: WT/Sham = 8, WT/CSI = 5, Rag1KO/Sham = 6, Rag1KO/CSI = 5, WT/Ctrl = 4, WT/CPA = 3, WT/GEM = 3, Rag1KO/Ctrl = 4, Rag1KO/CPA = 3, Rag1KO/GEM = 3.

To understand what was driving the immunological changes we observed following the administration of first line medulloblastoma treatments, and due to the inability to clearly delineate microglia from macrophages or monocytes through flow cytometry or IHC, we employed bulk RNA sequencing on medulloblastomas following control, sham, CSI, CPA, or GEM treatment. RNA sequencing was carried out on late-stage tumors, as tumors were too small to be isolated at earlier stages. As such, CSI treated samples were harvested 1-2 weeks after treatment cessation, while chemotherapy-treated tumors were harvested within 48-72 hours of drug administration. For scientific rigor, CSI treated mice were compared to sham controls, while chemotherapy-treated mice were compared to saline controls. Principal component analysis plots were used to visualize and identify whether samples clustered by treatment across both mouse strains (**Figure 6A**). Samples treated with CSI or sham did not cluster according to treatment or genetic background. For chemotherapy treated mice, Rag1KO samples clustered by treatment (Ctrl, CPA, GEM), whilst in WT mice, GEM clustered separately to CPA and Ctrl samples, which overlapped.

Through differential gene expression analysis, we identified 210 differentially expressed genes (DEGs) in WT CSI treated mice compared to sham (132 upregulated genes, 78 downregulated genes), while no significant changes in gene expression were observed in CSI treated Rag1KO samples compared to sham (**Figure 6B**, *left*). In WT mice, there were no DEGs in response to CPA treatment, whilst in Rag1KO CPA treated mice there were 3440 DEGs compared to Ctrl (2334 up, 1106 down). GEM treatment in WT mice induced 403 DEGs (245 up, 158 down) and 1857 DEGs in Rag1KO (934 up, 923 down) compared to Ctrl (**Figure 6B**).

We applied CIBERSORTx to determine the abundance of immune cell types within the bulk sequenced data and to clarify if these were altered following treatment. Immune signatures were very low, and only microglia, border associated macrophages (BAMs), and monocyte signatures were detected (**Figures 6C, D**), although these estimates were non-significant (P>0.05). CSI did not appear to significantly alter immune cell composition (**Figure 6C**). Despite the low signature values, when the computed cell fractions were compared from Rag1KO mice treated with CPA or GEM, microglia were decreased compared to control mice (P=2x10^-4^ and P=2x10^-5^, respectively), consistent with our flow cytometry findings (**Supplementary Figure 6**), and BAMs were significantly elevated (P=0.04 and P=1.8x10^-5^, respectively) (**Figure 6D**); no significant differences were observed in WT mice. Ultimately, immune cell composition could not be accurately quantified by deconvoluting our bulk RNA sequencing data likely due to the low abundance of immune cells relative to tumor cells in Myc/p53^DD^ tumors.

Pathway analysis also showed very few differentially expressed genes were associated with the immune system, owing to most genes being from tumor cells rather than from immune cells. Activated pathways in medulloblastomas in WT mice treated with CSI were associated with muscle contraction (P=3.70x10^-5^) and myogenesis (P=0.0143), whilst inhibited pathways were related to the neuronal system/ion channels (P=2.32x10^-4^). While there were no DEGs identified in CPA-treated medulloblastomas from WT mice, activated pathways in CPA-treated medulloblastomas from Rag1KO mice related to the neuronal system/ion channels (e.g. voltage ion channels, GABA receptors; P=1.94x10^-46^), signaling by GPCRs (P=7.26x10^-7^), MAPK signaling pathway (P=1.62x10^-5^), axon guidance (P=4.01x10^-5^), and hemostasis (P=3.6x10^-4^). Downregulated pathways were associated with ribosomal translation/gene expression (P=5.12x10^-13^), collagen biosynthesis/degradation & extracellular matrix (P<2.20x10^-8^) and cell cycle/proliferation (P=1.73x10^-7^). In response to GEM treatment, common activated pathways in both WT and Rag1KO mice were associated with metabolism (P<7.76x10^-5^), whilst ribosomal translation was unique to WT mice, and Hedgehog (P=0.0242), PPAR (P=0.0277) and interleukin signaling pathways (P=0.0281) were only activated in Rag1KO. Common downregulated pathways were associated with hemostasis (P<0.0229) and axon guidance (P<2.82x10^-4^); unique pathways in WT were linoleic acid metabolic (P=3.02x10^-4^) and p53 signaling (p=0.0449); inhibited pathways in Rag1KO mice only were developmental biology (P=1.70x10^-5^), MAPK signaling (P=0.00499), neuronal system (P=0.0277), cell cycle/proliferation (P=0.0262) and WNT signaling (P=0.0439).

Upstream regulator analysis was performed to identify putative drivers of the differentially expressed genes **(** **Supplementary Figure 7**). Overall, as expected given the above findings, the data showed limited immune system drivers and an array of activated chemical signatures, particularly in chemotherapy treated Rag1KO mice. In WT mice, the CSI response was driven by growth factor signaling (e.g. EGF, NRG1, ERBB3) and pro-inflammatory regulators (STAT3, PTGS2). In WT mice, GEM responses are driven by metabolic regulators (primarily lipid metabolism), while in Rag1KO mice, CPA and GEM responses are driven by chemical drivers, and cancer associated drivers were inhibited (MYC, SOX4, MYB).

## Discussion

Medulloblastoma is one of the most prevalent pediatric cancers. While 5-year survival rates are approximately 70%, particular genetic features are associated with worse prognosis and current clinical approaches require innovative rethinking to identify ways to improve outcomes for those patients. Immunotherapy has recently become a major focus of novel therapy development and there are multiple clinical trials that aim to increase immune cell recognition of medulloblastoma, including oncolytic viral therapy, cancer vaccines and immune checkpoint blockade [reviewed in (40)]. To develop future immunotherapy clinical trials for medulloblastoma that have a strong chance of improved efficacy with reduced adverse effects, a deeper understanding of the interactions between medulloblastoma and either brain resident immune cells or infiltrating immune cells is crucial.

While patient derived xenograft models provide insight into the genetic and molecular basis of medulloblastoma and are valuable in the investigation of molecularly targeted therapies, they do not allow for the study of the whole immune system due to their use of immunocompromised hosts. Ideally, preclinical models for testing novel immunotherapies must recapitulate orthotopic medulloblastoma as well as the immune microenvironment. Immune competent preclinical models are therefore required to complement patient-derived xenograft models in a robust and comprehensive preclinical drug testing pipeline. Here we used an immune-competent murine model of *Myc*-amplified Group 3 medulloblastoma to investigate changes in intracerebral immune cell populations induced by tumor growth, and for the first time describe the impact of several first line medulloblastoma therapies on the immune microenvironment. Moreover, we repeated this work in *Rag1* knockout mice lacking T and B cells to elucidate the role of adaptive immune cells in treatment response.

Our data show that adaptive immune cells account for a small proportion of immune cells in the brains of mice harboring Myc/p53^DD^ tumors. T cells account for between 10-15% of the immune population in Myc/p53^DD^ bearing brain, and B cells account for between 2-6% of the immune populations. Using *Rag1* deficient mice, we found that the adaptive immune system does not play a significant role in Myc/p53^DD^ tumor engraftment or growth, nor in treatment-mediated tumor control. Furthermore, both CSI and CPA were found to significantly deplete T cells in the brain. This, combined with the fact that lymphocytes are rare in human Group 3 medulloblastoma (41) and medulloblastoma patients have demonstrated low to undetectable levels of PD-L1 and PD-1 (42–44), suggests that T cell targeted antibody therapies, such as anti-PD-1, are unlikely to succeed in combination with radiotherapy or chemotherapy in medulloblastoma.

On the other hand, microglia dominate the immune milieu of the tumor bearing brain, accounting for between 50-65% of all immune cells. Further, we show that following either radiotherapy or chemotherapy, microglia remain the most abundant immune cell in the brain and thus present as a favorable target for immunotherapy in Group 3 medulloblastoma in combination with frontline therapies. Indeed, recent preclinical data has excitingly shown that treatments targeting myeloid cell immune checkpoints, such as the CD47-SIRPα axis, are highly effective in mouse models of medulloblastoma and other childhood brain cancers (45). Furthermore, it has been suggested that radiotherapy may synergize with monoclonal antibody therapies, on the basis that radiation enhances the visibility of medulloblastoma to the immune system [reviewed in (40)]. Recently, it has been shown that a single 10 Gy dose of radiation can induce an increase in tumor associated macrophages in SHH medulloblastoma (33), however, in our study using a clinically-relevant fractionated CSI protocol did not result in an increase in absolute counts of microglia nor macrophage at either acute or late-stage time points, and no increase in Iba1^+^ staining was observed in late-stage tumors after CSI (**Supplementary Figure 8**). For laboratory-based experiments to accurately inform new clinical trials in medulloblastoma, future work should aim to characterize the immune response to fractionated versus unfractionated radiotherapy doses across different medulloblastoma subtypes to better understand the optimal preclinical radiotherapy methods to apply.

In contrast to the effects of fractionated CSI, we found that CPA treatment reduced the proportions of MHC II^+^ microglia in mouse brain, suggesting this chemotherapy suppresses microglial function. We also examined the effects of GEM on immune cell populations in medulloblastoma, as this drug is currently being investigated as a first-line chemotherapy in Group 3 and Group 4 medulloblastoma (NCT01878617). The only cell population in the brain observed to be affected by GEM were neutrophils which were decreased following treatment. This was unexpected, as in previous cancer studies, GEM selectively depleted myeloid cells and B cells, in both a tumor microenvironment and in lymphoid organs (46, 47), and we observed depletion of T and B cells in the spleen of GEM treated WT mice (**Supplementary Figure 1**). Importantly, exposure to GEM can also increase tumor antigenicity through upregulation of MHC-I (48), inhibition of tumor-associated macrophages, and improving antigen cross-presentation (49, 50) to aid in immune stimulation and tumor elimination. Our data did not indicate that GEM treatment had these same effects in medulloblastoma, although given these previous studies, additional experiments to better delineate the effects of GEM on medulloblastoma-associated macrophages would be valuable.

Limitations of this study include the techniques and cellular markers used to identify immune populations from brains of mice with medulloblastoma. Flow cytometry is unable to determine the spatial interactions of microglia and infiltrating macrophages within murine medulloblastoma, and importantly our data suggest that the marker Tmem119, often used to distinguish microglia from macrophages, may be an unreliable cellular marker in this context. Using IHC, we observed an abundance of Iba1^+^ cells throughout medulloblastomas and an absence of Tmem119^+^ cells, suggesting intratumoral Iba1^+^ cells were not microglia. However, the absence of intratumoral Tmem119 staining is not consistent with our findings that 23.1% of intratumoral immune cells were CD45^int^, and presumed microglia. Given previous reports have shown microglia can upregulate CD45 expression in pathological conditions (36, 37), we hypothesize that bone marrow-derived macrophages do not account for the entirety of tumor-infiltrative Iba1^+^ Tmem119^-^ cells observed, and that microglia may downregulate Tmem119 as they enter medulloblastomas, suggesting a transition of these cells into a more macrophage-like state. While there are a number of methods that can clarify if these cells are activated microglia, such as MHC Class II, Sca-1 (51) or CD68 (52), these markers are also expressed on other myeloid cells, and would need to be assessed in combination with a microglia specific marker such as Sall1 (53).

Microglia and infiltrating macrophages are complex and dynamic in the context of cancer, and they cannot be easily delineated by simple markers. Indeed, recent single cell transcriptomics analysis of human samples revealed that an unexpectedly diverse spectrum of myeloid populations infiltrate medulloblastoma (41). Here, we set out to use transcriptomics to further characterize immune responses to treatment. However, we found that bulk RNA sequencing lacked sensitivity to probe the immunology of these tumors, largely due to the low abundance of intratumoral immune cells within this model of medulloblastoma. As a result, cell fractions were not significantly deconvoluted by CIBERSORTx and downstream comparisons of cell abundances should be carried out with caution. Our RNAseq data suggests that chemotherapy may alter the ratio of myeloid subsets (specifically microglia and BAMs) within the brain, but this requires additional experimental validation, particularly given the flow cytometry markers used here were not able to discriminate between these two cell types. Further examination using makers such as CD206, Siglec-H and CD38 that are expressed on BAMs and not microglia (51), as well as using histological techniques to define the locations of the chemotherapy-affected cells will be important to more accurately define the impacts of chemotherapy on the brain immune microenvironment especially given that the vasculature in the border regions would have different barrier properties compared to the blood-brain barrier of the parenchyma. Future work aimed at better understanding the immune cell dynamics within medulloblastomas may also consider first enriching for CD45^+^ cells prior to bulk RNA sequencing or employing single cell sequencing or single cell proteomics technologies to detect immune signatures in medulloblastoma tissue, though these methods are accompanied with the caveat of tissue processing induced artefacts [reviewed in (54)]. Our sequencing data was limited by small sample sizes in this exploratory study and, in the case of CSI-treated tumors, by the choice to sample later time points when tumors were larger, but possibly too late after treatment cessation to detect gene expression differences. Further, this study is limited by the intracranial implantation procedure disrupting the skull and meninges, which may mediate peripheral immune influx by promoting an inflammatory response and disrupting the blood brain barrier. Though we have found that the intracranial implantation procedure does not detectably change immune populations in the brain of non-tumor bearing animals (data not shown), genetically engineered spontaneous models of Group 3 medulloblastoma with an intact blood-brain barrier would improve the study of the tumor-immune microenvironment.

The study of the immune infiltrate within medulloblastoma is critical not only for the implementation of optimal radiotherapy and chemotherapy protocols, but very relevant to immunotherapy, a therapeutic modality with increasing use in oncology. Immunotherapies are not standard in the treatment of medulloblastoma, and clinical trials investigating the use of T-cell targeting immunotherapies have proven unsuccessful to date (40). This lack of success is likely a consequence of the immune microenvironment in pediatric brain tumors being very different from that of adult solid tumors in which immune-based therapies have proven successful. Overall, the most consistently abundant immune cell within this model of Group 3 medulloblastoma following radiotherapy or chemotherapy are myeloid cells, which we speculate are a mixture of both brain-resident and bone marrow-derived cells. Should myeloid cell-targeting therapies continue to be developed for medulloblastoma, future work should assess the impacts of radiotherapy and chemotherapy on microglial and macrophage activation and function, to dissect out mechanisms of treatment-induced changes and how this might impact the efficacy of immunotherapies. Building upon our study will be important to address this issue and will facilitate the rational selection of optimal immunotherapeutics for future medulloblastoma clinical trials.

## Data Availability Statement

The data presented in the study are deposited in the European Genome-Phenome Archive, accession numbers EGAS00001005846 and EGAS00001005847.

## Ethics Statement

All animal studies in this work were reviewed and approved by the Animal Ethics Committee of the Telethon Kids Institute.

## Author Contributions

ZA, CG, MH, RE, and NG designed the experiments. ZA, CG, MA, AJ, MK, and RE performed experiments or analyzed data. ZA, CG, MA, AJ, MH, RW-R, NG, and RE were involved in data discussion, drafting, and editing the manuscript. All authors contributed to the article and approved the submitted version.

## Funding

This research was funded by The Pirate Ship Foundation. RNA sequencing experiments were partially funded through the Australian Lions Children’s Cancer Research Fund and the Stan Perron Charitable Foundation. RE has support from a Cancer Council of Western Australia Research Fellowship and a Brainchild Fellowship from the Pirate Ship Foundation. NG is supported by the Stan Perron Chair of Paediatric Haematology and Oncology. ZA is supported by a Richard Walter Gibbon Medical Research Scholarship and an Australian Government Research Training Program Scholarship at the University of Western Australia. MA and CG were supported by an Australian Postgraduate Award from the Australian Government. AJ is supported by the Stan Perron Foundation, Australian Lions Children’s Cancer Research Foundation and Channel 7 Telethon Trust. RW-R is supported by funds from the National Cancer Institute (2R01 CA159859, P30 CA30199), the National Institute of Neurological Disorders and Stroke (R35 NS122339, R01 NS096368) and the V Foundation for Cancer Research (D2018-021).

## Conflict of Interest

The authors declare that the research was conducted in the absence of any commercial or financial relationships that could be construed as a potential conflict of interest.

## Publisher’s Note

All claims expressed in this article are solely those of the authors and do not necessarily represent those of their affiliated organizations, or those of the publisher, the editors and the reviewers. Any product that may be evaluated in this article, or claim that may be made by its manufacturer, is not guaranteed or endorsed by the publisher.

## References

[1] Northcott PA Robinson GW Kratz CP Mabbott DJ Pomeroy SL Clifford SC. Medulloblastoma. Nat Rev Dis Primers (2019) 5(1):11. doi: 10.1038/s41572-019-0063-6 30765705

[2] Cavalli FMG Remke M Rampasek L Peacock J Shih DJH Luu B. Intertumoral Heterogeneity Within Medulloblastoma Subgroups. Cancer Cell (2017) 31(6):737–54.e6. doi: 10.1016/j.ccell.2017.05.005 28609654 PMC6163053

[3] Schwalbe EC Lindsey JC Nakjang S Crosier S Smith AJ Hicks D. Novel Molecular Subgroups for Clinical Classification and Outcome Prediction in Childhood Medulloblastoma: A Cohort Study. Lancet Oncol (2017) 18(7):958–71. doi: 10.1016/S1470-2045(17)30243-7 PMC5489698 28545823

[4] Leary SES Olson JM. The Molecular Classification of Medulloblastoma. Curr Opin Pediatr (2012) 24(1):33–9. doi: 10.1097/MOP.0b013e32834ec106 PMC3348176 22189395

[5] Hill RM Richardson S Schwalbe EC Hicks D Lindsey JC Crosier S. Time, Pattern, and Outcome of Medulloblastoma Relapse and Their Association With Tumour Biology at Diagnosis and Therapy: A Multicentre Cohort Study. Lancet Child Adolesc Health (2020) 4(12):865–74. doi: 10.1016/S2352-4642(20)30246-7 PMC7671998 33222802

[6] Kool M Korshunov A Remke M Jones DT Schlanstein M Northcott PA. Molecular Subgroups of Medulloblastoma: An International Meta-Analysis of Transcriptome, Genetic Aberrations, and Clinical Data of WNT, SHH, Group 3, and Group 4 Medulloblastomas. Acta Neuropathol (2012) 123(4):473–84. doi: 10.1007/s00401-012-0958-8 PMC3306778 22358457

[7] Richardson S Hill RM Kui C Lindsey JC Grabovksa Y Keeling C. Emergence and Maintenance of Actionable Genetic Drivers at Medulloblastoma Relapse. Neuro Oncol (2022) 24(1):153–65. doi: 10.1093/neuonc/noab178 PMC8730763 34272868

[8] Koschmann C Bloom K Upadhyaya S Geyer JR Leary SES. Survival After Relapse of Medulloblastoma. J Pediatr Hematol/Oncol (2016) 38(4):269–73. doi: 10.1097/MPH.0000000000000547 26907655

[9] Sabel M Fleischhack G Tippelt S Gustafsson G Doz F Kortmann R. Relapse Patterns and Outcome After Relapse in Standard Risk Medulloblastoma: A Report From the HIT-SIOP-PNET4 Study. J Neuro-Oncol (2016) 129(3):515–24. doi: 10.1007/s11060-016-2202-1 PMC5020107 27423645

[10] Pei Y Moore CE Wang J Tewari AK Eroshkin A Cho YJ. An Animal Model of MYC-Driven Medulloblastoma. Cancer Cell (2012) 21(2):155–67. doi: 10.1016/j.ccr.2011.12.021 PMC3285431 22340590

[11] Mombaerts P Iacomini J Johnson RS Herrup K Tonegawa S Papaioannou VE. RAG-1-Deficient Mice Have No Mature B and T Lymphocytes. Cell (1992) 68(5):869–77. doi: 10.1016/0092-8674(92)90030-G 1547488

[12] Endersby R Zhu X Hay N Ellison DW Baker SJ. Nonredundant Functions for Akt Isoforms in Astrocyte Growth and Gliomagenesis in an Orthotopic Transplantation Model. Cancer Res (2011) 71(12):4106–16. doi: 10.1158/0008-5472.CAN-10-3597 PMC3118569 21507933

[13] Feddersen TV Rowshanfarzad P Abel TN Ebert MA. Commissioning and Performance Characteristics of a Pre-Clinical Image-Guided Radiotherapy System. Australas Phys Eng Sci Med (2019) 42(2):541–51. doi: 10.1007/s13246-019-00755-4 PMC6557883 30989595

[14] van Hoof SJ Granton PV Verhaegen F. Development and Validation of a Treatment Planning System for Small Animal Radiotherapy: Smart-Plan. Radiother Oncol (2013) 109(3):361–6. doi: 10.1016/j.radonc.2013.10.003 24183860

[15] Smith SMC Bianski BM Orr BA Harknett G Onar-Thomas A Gilbertson RJ. Preclinical Modeling of Image-Guided Craniospinal Irradiation for Very-High-Risk Medulloblastoma. Int J Radiat Oncol Biol Phys (2019) 103(3):728–37. doi: 10.1016/j.ijrobp.2018.10.015 PMC6421074 30366006

[16] Martin M. Cutadapt Removes Adapter Sequences From High-Throughput Sequencing Reads. EMBnetjournal (2011) 17(1):10. doi: 10.14806/ej.17.1.200

[17] Andrews S FASTQC. A Quality Control Tool for High Throughput Sequence Data (2010). Available at: https://www.bioinformatics.babraham.ac.uk/projects/fastqc/.

[18] Lassmann T Hayashizaki Y Daub CO. Samstat: Monitoring Biases in Next Generation Sequencing Data. Bioinformatics (2011) 27(1):130–1. doi: 10.1093/bioinformatics/btq614 PMC3008642 21088025

[19] Kim D Langmead B Salzberg SL. HISAT: A Fast Spliced Aligner With Low Memory Requirements. Nat Methods (2015) 12(4):357–60. doi: 10.1038/nmeth.3317 PMC4655817 25751142

[20] Lawrence M Huber W Pagès H Aboyoun P Carlson M Gentleman R. Software for Computing and Annotating Genomic Ranges. PloS Comput Biol (2013) 9(8):e1003118. doi: 10.1371/journal.pcbi.1003118 23950696 PMC3738458

[21] Risso D Ngai J Speed TP Dudoit S. Normalization of RNA-Seq Data Using Factor Analysis of Control Genes or Samples. Nat Biotechnol (2014) 32(9):896–902. doi: 10.1038/nbt.2931 25150836 PMC4404308

[22] Newman AM Steen CB Liu CL Gentles AJ Chaudhuri AA Scherer F. Determining Cell Type Abundance and Expression From Bulk Tissues With Digital Cytometry. Nat Biotechnol (2019) 37(7):773–82. doi: 10.1038/s41587-019-0114-2 PMC6610714 31061481

[23] Van Hove H Martens L Scheyltjens I De Vlaminck K Pombo Antunes AR De Prijck S. A Single-Cell Atlas of Mouse Brain Macrophages Reveals Unique Transcriptional Identities Shaped by Ontogeny and Tissue Environment. Nat Neurosci (2019) 22(6):1021–35. doi: 10.1038/s41593-019-0393-4 31061494

[24] Robinson MD McCarthy DJ Smyth GK. Edger: A Bioconductor Package for Differential Expression Analysis of Digital Gene Expression Data. Bioinformatics (2010) 26(1):139–40. doi: 10.1093/bioinformatics/btp616 PMC2796818 19910308

[25] Breuer K Foroushani AK Laird MR Chen C Sribnaia A Lo R. Innatedb: Systems Biology of Innate Immunity and Beyond–Recent Updates and Continuing Curation. Nucleic Acids Res (2013) 41(Database issue):D1228–D33. doi: 10.1093/nar/gks1147 PMC3531080 23180781

[26] Krämer A Green J Pollard J Jr. Tugendreich S. Causal Analysis Approaches in Ingenuity Pathway Analysis. Bioinformatics (2014) 30(4):523–30. doi: 10.1093/bioinformatics/btt703 PMC3928520 24336805

[27] Bender R Lange S. Adjusting for Multiple Testing—When and How? J Clin Epidemiol (2001) 54(4):343–9. doi: 10.1016/S0895-4356(00)00314-0 11297884

[28] Martin AM Raabe E Eberhart C Cohen KJ. Management of Pediatric and Adult Patients With Medulloblastoma. Curr Treat Options Oncol (2014) 15(4):581–94. doi: 10.1007/s11864-014-0306-4 PMC4216607 25194927

[29] Pham CD Flores C Yang C Pinheiro EM Yearley JH Sayour EJ. Differential Immune Microenvironments and Response to Immune Checkpoint Blockade Among Molecular Subtypes of Murine Medulloblastoma. Clin Cancer Res (2016) 22(3):582–95. doi: 10.1158/1078-0432.CCR-15-0713 PMC4922139 26405194

[30] Sedgwick JD Schwender S Imrich H Dorries R Butcher GW Ter Meulen V. Isolation and Direct Characterization of Resident Microglial Cells From the Normal and Inflamed Central Nervous System. Proc Natl Acad Sci (1991) 88(16):7438–42. doi: 10.1073/pnas.88.16.7438 PMC52311 1651506

[31] O’Koren EG Mathew R Saban DR. Fate Mapping Reveals That Microglia and Recruited Monocyte-Derived Macrophages Are Definitively Distinguishable by Phenotype in the Retina. Sci Rep (2016) 6(1):20636. doi: 10.1038/srep20636 26856416 PMC4746646

[32] Wyss-Coray T Mucke L. Inflammation in Neurodegenerative Disease–A Double-Edged Sword. Neuron (2002) 35(3):419–32. doi: 10.1016/S0896-6273(02)00794-8 12165466

[33] Dang MT Gonzalez MV Gaonkar KS Rathi KS Young P Arif S. Macrophages in SHH Subgroup Medulloblastoma Display Dynamic Heterogeneity That Varies With Treatment Modality. Cell Rep (2021) 34(13):108917. doi: 10.1016/j.celrep.2021.108917 33789113 PMC10450591

[34] Galluzzi L Buque A Kepp O Zitvogel L Kroemer G. Immunological Effects of Conventional Chemotherapy and Targeted Anticancer Agents. Cancer Cell (2015) 28(6):690–714. doi: 10.1016/j.ccell.2015.10.012 26678337

[35] Ahlmann M Hempel G. The Effect of Cyclophosphamide on the Immune System: Implications for Clinical Cancer Therapy. Cancer Chemother Pharmacol (2016) 78(4):661–71. doi: 10.1007/s00280-016-3152-1 27646791

[36] Honarpisheh P Lee J Banerjee A Blasco-Conesa MP Honarpisheh P D’Aigle J. Potential Caveats of Putative Microglia-Specific Markers for Assessment of Age-Related Cerebrovascular Neuroinflammation. J Neuroinflamm (2020) 17(1):366. doi: 10.1186/s12974-020-02019-5 PMC7709276 33261619

[37] Muller A Brandenburg S Turkowski K Muller S Vajkoczy P. Resident Microglia, and Not Peripheral Macrophages, Are the Main Source of Brain Tumor Mononuclear Cells. Int J Cancer (2015) 137(2):278–88. doi: 10.1002/ijc.29379 25477239

[38] Sasaki Y Ohsawa K Kanazawa H Kohsaka S Imai Y. Iba1 Is an Actin-Cross-Linking Protein in Macrophages/Microglia. Biochem Biophys Res Commun (2001) 286(2):292–7. doi: 10.1006/bbrc.2001.5388 11500035

[39] Bennett ML Bennett FC Liddelow SA Ajami B Zamanian JL Fernhoff NB. New Tools for Studying Microglia in the Mouse and Human CNS. Proc Natl Acad Sci USA (2016) 113(12):E1738–46. doi: 10.1073/pnas.1525528113 PMC4812770 26884166

[40] Kabir TF Kunos CA Villano JL Chauhan A. Immunotherapy for Medulloblastoma: Current Perspectives. Immunotarg Ther (2020) 9:57–77. doi: 10.2147/ITT.S198162 PMC7182450 32368525

[41] Riemondy KA Venkataraman S Willard N Nellan A Sanford B Griesinger AM. Neoplastic and Immune Single Cell Transcriptomics Define Subgroup-Specific Intra-Tumoral Heterogeneity of Childhood Medulloblastoma. Neuro Oncol (2021) 24(2):273–86. doi: 10.1093/neuonc/noab135 PMC8804892 34077540

[42] Vermeulen JF Van Hecke W Adriaansen EJM Jansen MK Bouma RG Villacorta Hidalgo J. Prognostic Relevance of Tumor-Infiltrating Lymphocytes and Immune Checkpoints in Pediatric Medulloblastoma. Oncoimmunology (2018) 7(3):e1398877. doi: 10.1080/2162402X.2017.1398877 29399402 PMC5790383

[43] Hwang K Koh EJ Choi EJ Kang TH Han JH Choe G. PD-1/PD-L1 and Immune-Related Gene Expression Pattern in Pediatric Malignant Brain Tumors: Clinical Correlation With Survival Data in Korean Population. J Neurooncol (2018) 139(2):281–91. doi: 10.1007/s11060-018-2886-5 29730815

[44] Martin AM Nirschl CJ Polanczyk MJ Bell WR Nirschl TR Harris-Bookman S. PD-L1 Expression in Medulloblastoma: An Evaluation by Subgroup. Oncotarget (2018) 9(27):19177–91. doi: 10.18632/oncotarget.24951 PMC5922386 29721192

[45] Gholamin S Mitra SS Feroze AH Liu J Kahn SA Zhang M. Disrupting the CD47-Sirpα Anti-Phagocytic Axis by a Humanized Anti-CD47 Antibody Is an Efficacious Treatment for Malignant Pediatric Brain Tumors. Sci Trans Med (2017) 9(381):eaaf2968. doi: 10.1126/scitranslmed.aaf2968 28298418

[46] Nowak AK Robinson BWS Lake RA. Gemcitabine Exerts a Selective Effect on the Humoral Immune Response. Cancer Res (2002) 62(8):2353. 11956096

[47] Suzuki E Kapoor V Jassar AS Kaiser LR Albelda SM. Gemcitabine Selectively Eliminates Splenic Gr-1+/CD11b+ Myeloid Suppressor Cells in Tumor-Bearing Animals and Enhances Antitumor Immune Activity. Clin Cancer Res (2005) 11(18):6713–21. doi: 10.1158/1078-0432.CCR-05-0883 16166452

[48] McDonnell AM Lesterhuis WJ Khong A Nowak AK Lake RA Currie AJ. Tumor-Infiltrating Dendritic Cells Exhibit Defective Cross-Presentation of Tumor Antigens, But Is Reversed by Chemotherapy. Eur J Immunol (2015) 45(1):49–59. doi: 10.1002/eji.201444722 25316312

[49] Homma Y Taniguchi K Murakami T Nakagawa K Nakazawa M Matsuyama R. Immunological Impact of Neoadjuvant Chemoradiotherapy in Patients With Borderline Resectable Pancreatic Ductal Adenocarcinoma. Ann Surg Oncol (2014) 21(2):670–6. doi: 10.1245/s10434-013-3390-y 24310792

[50] Eriksson E Wenthe J Irenaeus S Loskog A Ullenhag G. Gemcitabine Reduces Mdscs, Tregs and Tgfβ-1 While Restoring the Teff/Treg Ratio in Patients With Pancreatic Cancer. J Trans Med (2016) 14(1):282. doi: 10.1186/s12967-016-1037-z PMC5041438 27687804

[51] Mrdjen D Pavlovic A Hartmann FJ Schreiner B Utz SG Leung BP. High-Dimensional Single-Cell Mapping of Central Nervous System Immune Cells Reveals Distinct Myeloid Subsets in Health, Aging, and Disease. Immunity (2018) 48(2):380–95 e6. doi: 10.1016/j.immuni.2018.01.011 29426702

[52] Stankov A Belakaposka-Srpanova V Bitoljanu N Cakar L Cakar Z Rosoklija G. Visualisation of Microglia With the Use of Immunohistochemical Double Staining Method for CD-68 and Iba-1 of Cerebral Tissue Samples in Cases of Brain Contusions. Pril (Makedon Akad Nauk Umet Odd Med Nauki) (2015) 36(2):141–5. doi: 10.1515/prilozi-2015-0062 27442380

[53] Buttgereit A Lelios I Yu X Vrohlings M Krakoski NR Gautier EL. Sall1 is a Transcriptional Regulator Defining Microglia Identity and Function. Nat Immunol (2016) 17(12):1397–406. doi: 10.1038/ni.3585 27776109

[54] Machado L Relaix F Mourikis P. Stress Relief: Emerging Methods to Mitigate Dissociation-Induced Artefacts. Trends Cell Biol (2021) 31(11):888–97. doi: 10.1016/j.tcb.2021.05.004 34074577

