# Supplementary material for: Conventional Therapies Deplete Brain-Infiltrating Adaptive Immune Cells in a Mouse Model of Group 3 Medulloblastoma Implicating Myeloid Cells as Favorable Immunotherapy Targets
Source: Front Immunol. 2022 Mar 3;13:837013. doi: 10.3389/fimmu.2022.837013 (PMC8928748; doi:10.3389/fimmu.2022.837013)
Supplement: Supplementary file 1 [file DataSheet_1.pdf]

Supplementary Methods

Spleen single cell suspensions for flow cytometry

Spleens were dissociated in 5 mL of digestion buffer buffer (100 U/mL Collagenase IV (Life Technologies), 10 U/mL DNAse (Sigma-Aldrich) in Hank's balanced salt solution (HBSS, Gibco)) in a gentleMACS C tube on a gentleMACS Octo Dissociator on the preset mouse spleen program (m_spleen_01). Digestion was halted with the addition of 10 mL cold FACS buffer (2% fetal calf serum, 5 mM EDTA in HBSS) and the suspension was filtered through a 100 µm filter. Red blood cells were lysed with red blood cell lysis solution (Miltenyi), cells were resuspended in 10 mL FACS buffer, strained through a 30 µm filter (Miltenyi) and resuspended in DBPS for staining.

Flow cytometry antibody staining

Single cells were suspended in DPBS and stained with the viability dye (BD Bioscience Cat #564997, 1:20,000) for 20 minutes at room temperature in the dark. Cells were washed with DPBS and resuspended in FACS buffer, Fc receptors were blocked with Mouse BD Fc Block (BD Bioscience Cat #553142) and cells stained with the surface marker antibody panel (Supplementary Table 2) for 30 minutes at 4 °C in the dark. Cells were washed and resuspended in FACS buffer for sample acquisition.

Supplementary Table 1. Flow cytometry antibody panel used to stain brain and spleen single cell suspensions

Antibody-Fluorophore	Manufacturer	Catalogue number	Dilution
CD45-BV421	BD Bioscience	#563890	1:300
IAIE-BV510	Biolegend	#107635	1:300
CD11b-BV605	Biolegend	#101257	1:100
CD4-BV650	BD Bioscience	#563747	1:200
CD8a-BV711	BD Bioscience	#563046	1:100
NK1.1-BV785	Biolegend	#108749	1:100
B220-PerCP Cy5.5	BD Bioscience	#552771	1:200
F4/80-PE	BD Bioscience	#565410	1:100
CD3e-PECF594	BD Bioscience	#562286	1:200
CD19 PECy7	BD Bioscience	#552854	1:400
CD11c-APC	BD Bioscience	#550261	1:200
Ly6G-APC Cy7	BD Bioscience	#560600	1:100

Supplementary Table 2. Markers examined using flow cytometry to identify specific immune cell populations.

Cell type	Markers
Innate	
Microglia	$CD45^{intermediate}$, $CD11b^{+}$
$MHC\ II^{high}$ microglia (activated)	$CD45^{intermediate}$, $CD11b^{+}$, $MHC\ II^{high}$
Neutrophils	$CD45^{high}$, $CD11b^{+}$, $Ly6G^{+}$
Classic dendritic cells (cDC)	
$CD11b^{+}$ cDCs	$CD45^{high}$, $CD11c^{+}$, $MHC\ II^{+}$, $CD11b^{+}$, $CD8^{-}$
$CD8^{+}$ cDCs	$CD45^{high}$, $CD11c^{+}$, $MHC\ II^{+}$, $CD11b^{-}$, $CD8^{+}$
Natural killer cells (NK)	$CD45^{high}$, $CD11b^{+}$, $NK1.1^{+}$
Monocytes	$CD45^{high}$, $CD11b^{+}$, $F4/80^{+}$, FSC^{low}
Macrophages	$CD45^{high}$, $CD11b^{+}$, $F4/80^{+}$, FSC^{high}
Adaptive	
T cells	
$CD4^{+}$ T cells	$CD45^{high}$, $CD3^{+}$, $CD4^{+}$, $CD8^{-}$
$CD8^{+}$ T cells	$CD45^{high}$, $CD3^{+}$, $CD4^{-}$, $CD8^{+}$
B cells	$CD45^{high}$, $CD19^{+}$, $B220^{+}$

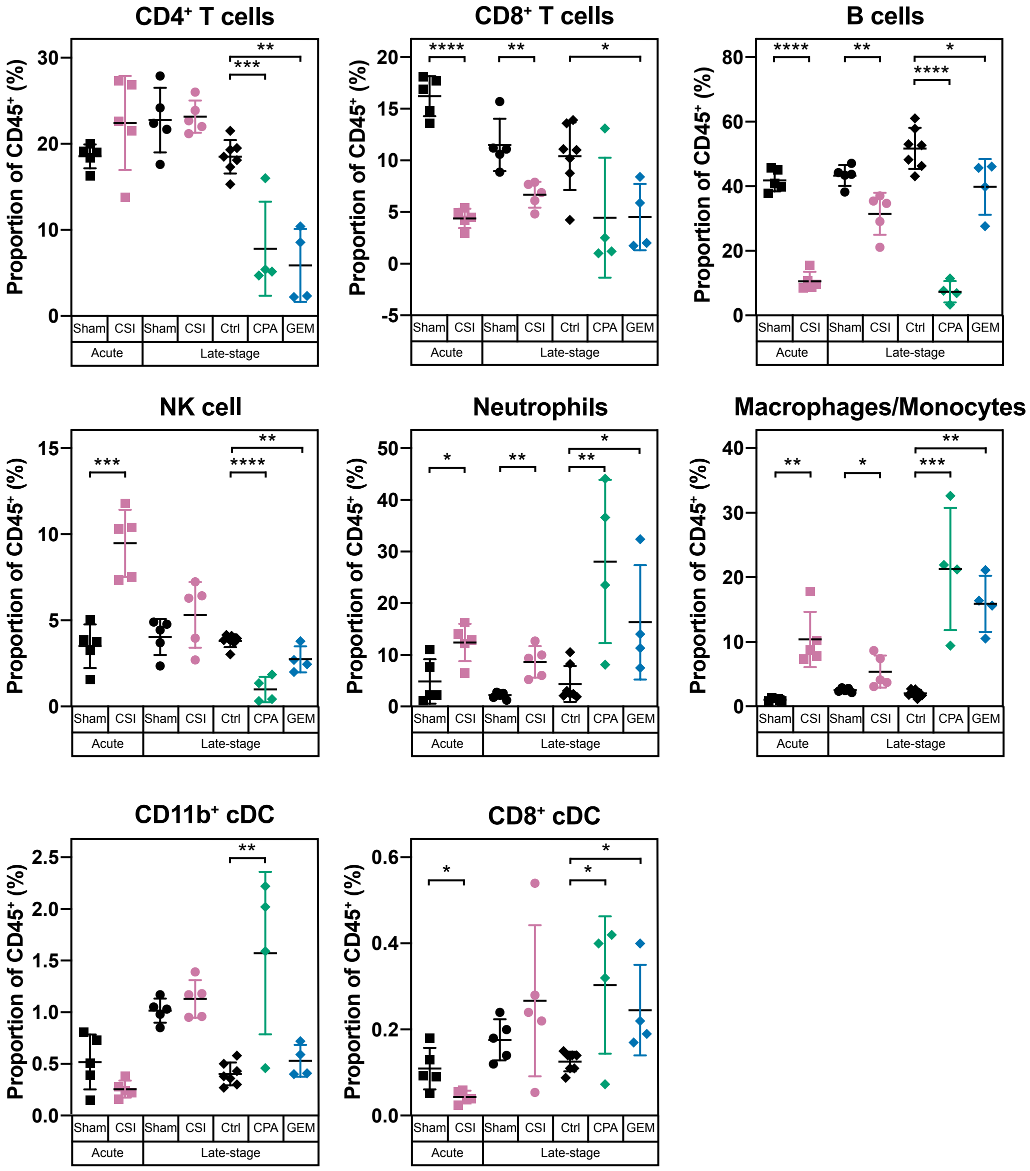

Figure S1. Conventional medulloblastoma treatments alter immune populations in the spleens of C57Bl/6J WT mice harboring Myc/$p53^{DD}$ tumors. Immune cell populations (shown as percentage of all $CD45^+$ cells) from the spleens of C57Bl/6J WT mice harboring Myc/$p53^{DD}$ medulloblastoma were treated with, from left to right, either sham (*black squares*, n=5) or CSI (*pink squares*, n=5) and harvested 24 hours after the final dose (labelled "Acute"), sham (*black circles*, n=5) or CSI (*pink circles*, n=5) and harvested upon the development of tumor-related morbidity (labelled "Late-stage") or after treatment with saline ("Ctrl", *black diamonds*, n=7), CPA (*green diamonds*, n=4), or GEM (*blue diamonds*, n=4) and harvested upon the development of tumor-related morbidity (also labelled "Late-stage"). See Figure 1 in the main text for details of treatment schedules. CSI, CPA and GEM all significantly depleted B cells and $CD8^+$ T cells, while only CPA and GEM significantly depleted $CD4^+$ T cells and NK cells. The depletion of B and $CD8^+$ T cells in CSI treated mice persisted until late-stage tumor development (1-2 weeks following treatment cessation). The depletion of $CD8^+$ T cells and B cells by CSI resulted in a proportional increase in NK cells, neutrophils, and macrophages/monocytes in the spleen. Similarly, a proportional increase in neutrophils and macrophages/monocytes was observed following CPA and GEM treatment, as well as a proportional increase in cDC populations. Horizontal lines indicate the mean and error bars indicate standard deviation. Comparisons by *t test* are shown, with statistically significant differences indicated (* $P<0.05$; ** $P<0.01$; *** $P<0.001$; **** $P<0.0001$).

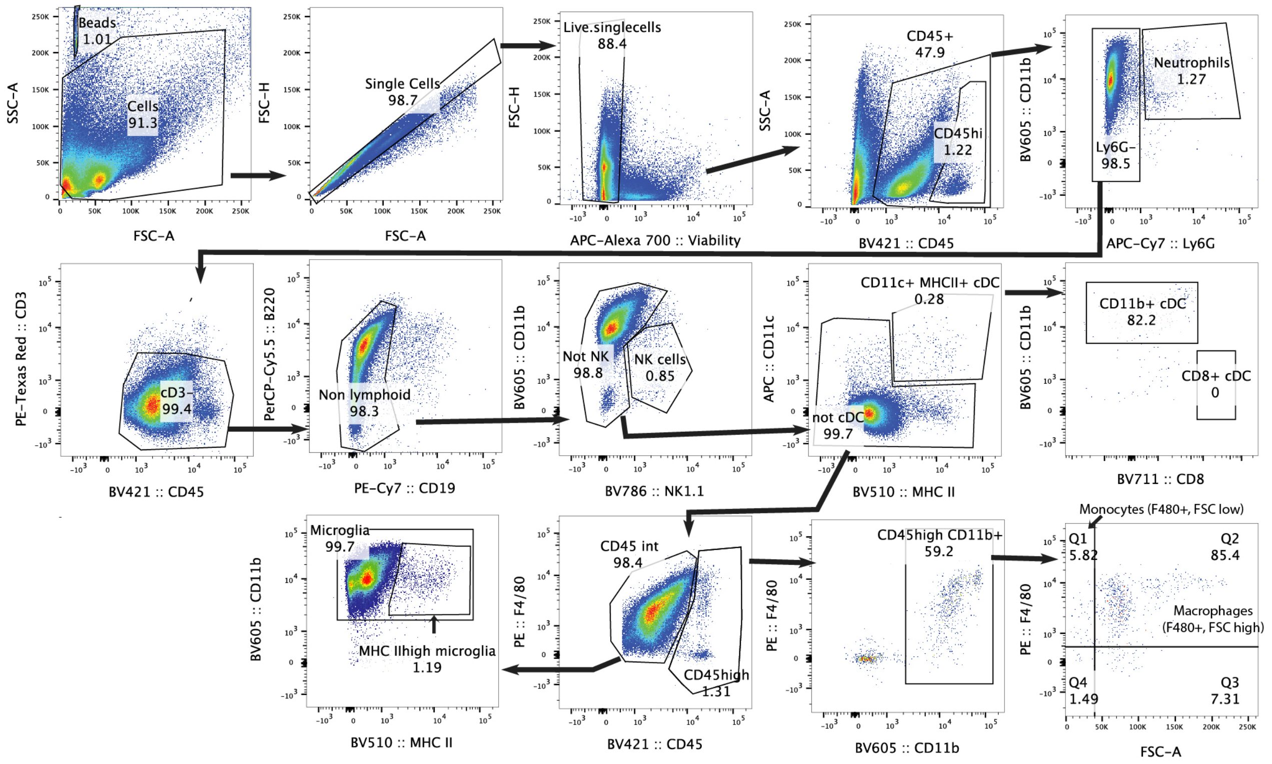

Figure S2. Gating strategy for flow cytometry analaysis of Rag1KO mouse brain.
Representative plots of the gating strategy used to identify the indicated immune populations from C57Bl/6J Rag1KO brain are shown.

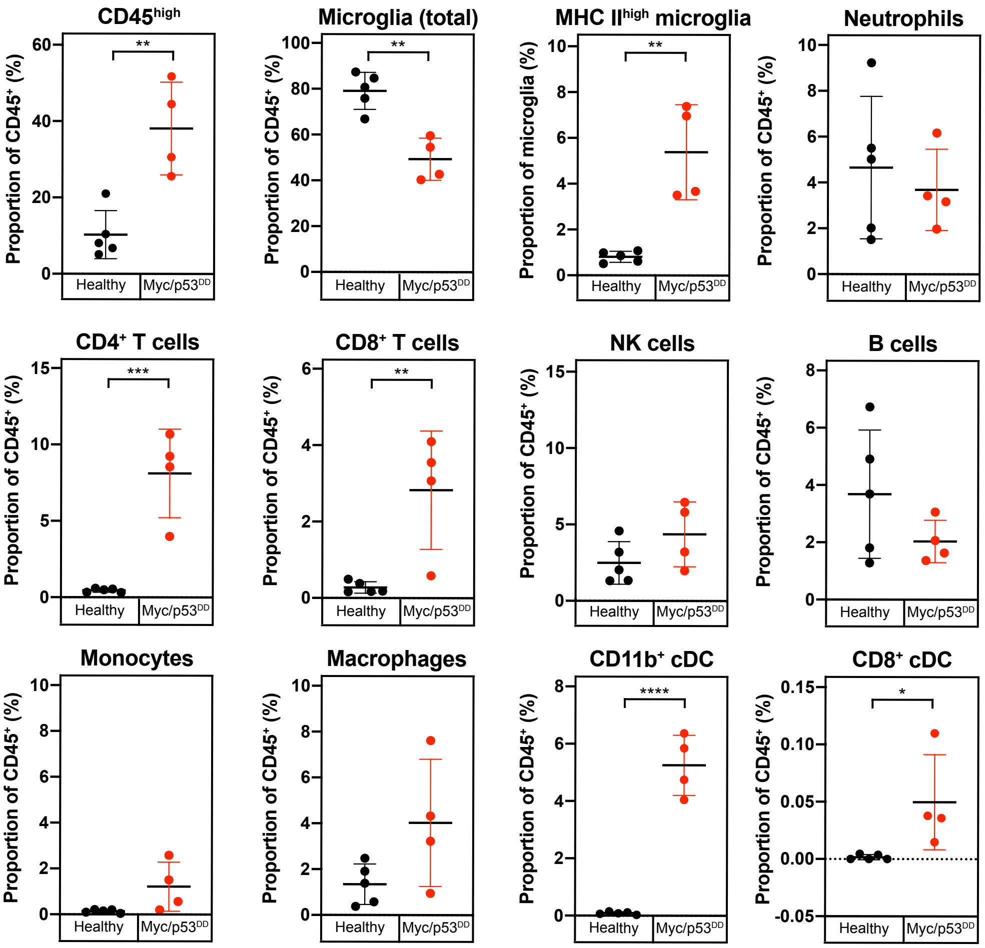

Figure S3. Growth of Myc/p53^{DD} medulloblastoma alters the ratios of immune cell populations in the brain of C57Bl/6J WT mice. Immune cell populations (shown as a percentage of all CD45^{+} cells) from healthy C57Bl/6J WT sham treated mice (*black,* n=5) were compared with sham treated WT brains harboring medulloblastoma using flow cytometry of brains harvested when mice developed tumor-related morbidity (*red*, n=4). The proportion of total immune cells which were peripheral immune cells (CD45^{high}) was significantly elevated in tumor bearing brains. Specifically, CD4^{+} T cells, CD8^{+} T cells, CD11b^{+} cDC and CD8^{+} cDC were all elevated in tumor bearing brains. This influx of peripheral cells resulted in a proportional decrease in microglia, but an increase in activated microglia (MHC II^{high}) was observed in tumor bearing brains. Horizontal lines indicate the mean and error bars indicate standard deviation. Comparisons by *t test* are shown, with statistically significant differences indicated (* $P<0.05$; ** $P<0.01$; *** $P<0.001$; **** $P<0.0001$).

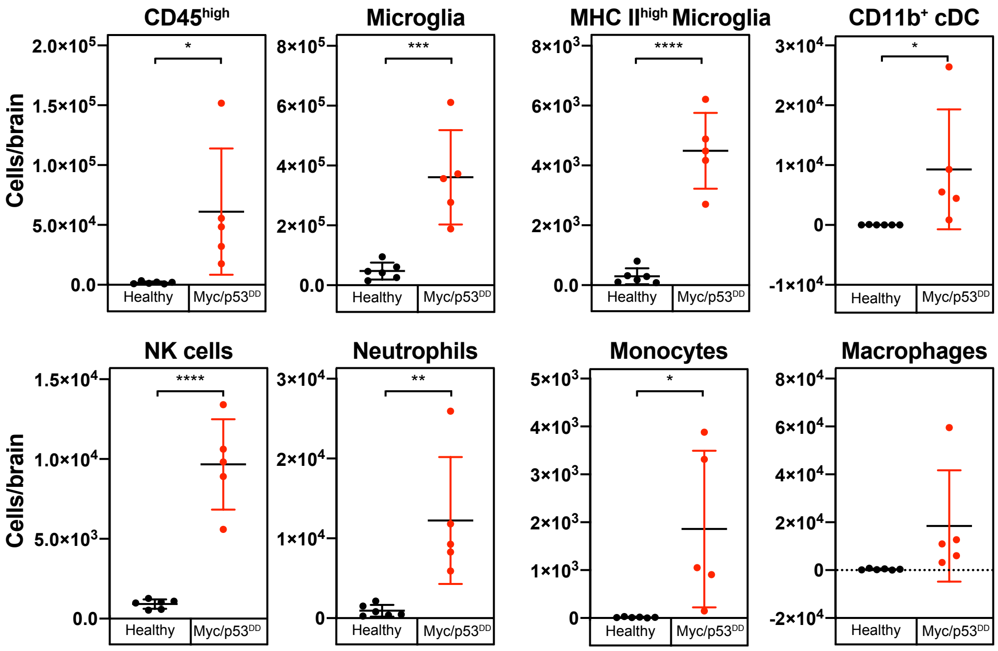

Figure S4. Myc/p53^{DD} medulloblastoma growth in Rag1KO mouse brain induces an increase in the absolute numbers of peripheral and resident immune cells. Sham treated Rag1KO mice bearing Myc/p53^{DD} tumors (*red*, n=5) have significantly higher overall counts of infiltrative (CD45^{high}) and resident (CD45^{int}) immune cells than sham treated healthy Rag1KO brains (*black*, n=6). Microglia (total and activated), CD11b^{+} cDCs, NK cells, neutrophils, and monocytes are elevated by the presence of Myc/p53^{DD} tumors. Macrophages were unaltered, CD8^{+} cDCs were not detected in Rag1KO mice. Horizontal lines indicate the mean and error bars indicate standard deviation. Comparison by *t test* are shown, with statistically significant differences indicated (* P<0.05; ** P<0.01; *** P<0.001; **** P<0.0001)

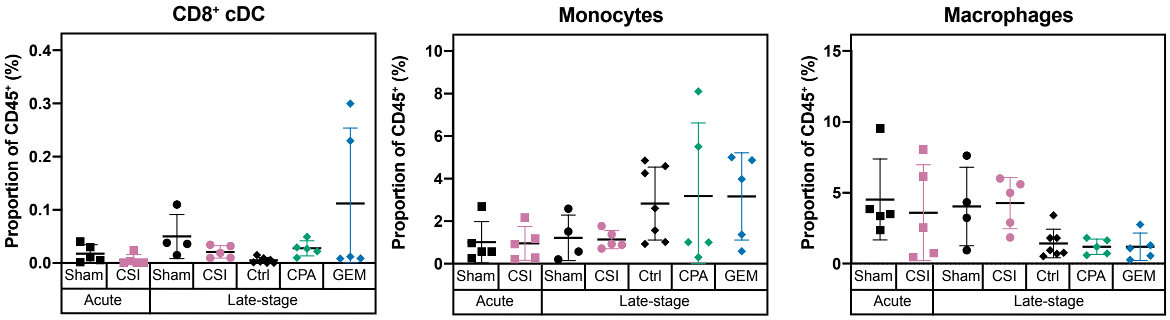

Figure S5. Radiotherapy and chemotherapy do not alter CD8$^+$ cDC, monocyte, and macrophage populations in WT mouse brain. Immune cell populations (shown as percentage of all CD45$^+$ cells) from C57Bl/6J WT mouse brain harboring Myc/p53^{DD} medulloblastoma were treated with either sham (*black squares*, n=5) or CSI (*pink squares,* n=5) and harvested 24 hours after the tenth dose (labelled "Acute"), sham (*black circles*, n=4) or CSI (*pink circles,* n=5) harvested upon the development of tumor-related morbidity (labeled "Late-stage"), or after treatment with saline (Ctrl, *black diamonds*, n=7), CPA (*green diamonds*, n=5), or GEM (*blue diamonds,* n=5) (also labelled "Late-stage"). See Figure 1 for details of treatment schedules. CD8$^+$ cDC's account for a very small proportion of immune cells in the brain and are unchanged following treatment. The abundance of monocytes and macrophage was unchanged following treatment. Horizontal lines indicate the mean and error bars indicate standard deviation. Each treatment group was compared to the appropriate treatment and time-point matched control by *t test*.

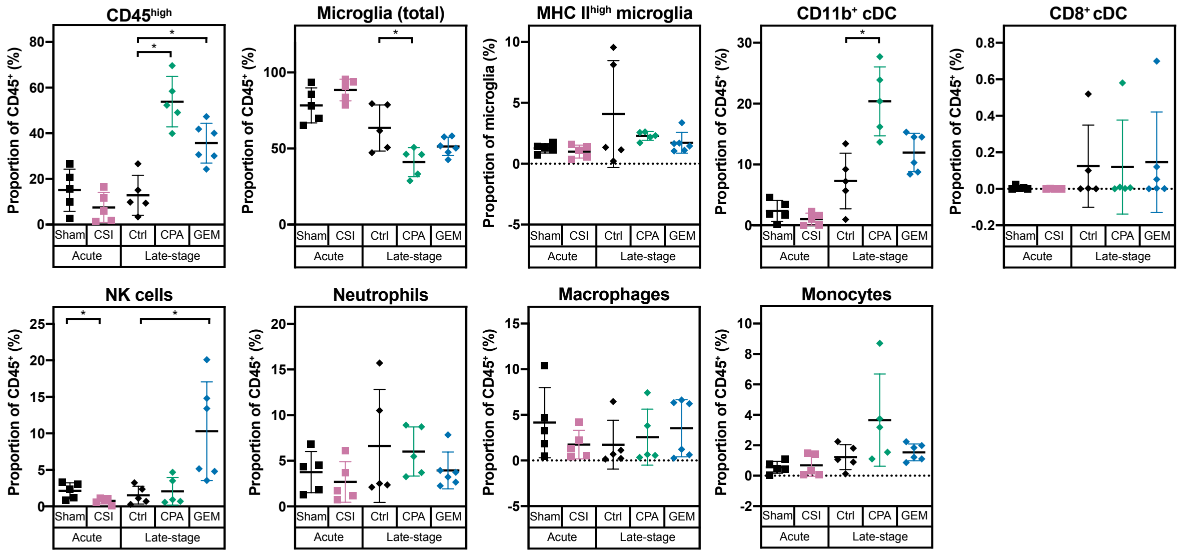

Figure S6. Radiotherapy and chemotherapy alter immune populations in brains of Rag1KO mice. Immune cell populations (shown as percentage of all $CD45^+$ cells) from C57Bl/6J Rag1KO mouse brain harboring Myc/$p53^{DD}$ medulloblastoma were treated with either sham (*black squares*, n=5) or CSI (*pink squares*, n=5) and harvested 24 hours after the final dose (labelled "Acute"), or after treatment with saline (Ctrl, *black diamonds,* n=7), CPA (*green diamonds*, n=5), or GEM (*blue diamonds,* n=6) (labelled "Late-stage"). See Figure 1 in the main text for details of treatment schedules. The proportion of peripheral immune cells ($CD45^{high}$) were significantly increased after both GEM and CPA treatment, but a decrease in microglia was only detected in CPA treated mice. CPA resulted in the recruitment of $CD11b^+$ cDC, while GEM resulted in the recruitment of NK cells in the brain. CSI resulted only in the depletion of NK cells. Horizontal lines indicate the mean and error bars indicate standard deviation. Each treatment group was compared to the appropriate treatment and time-point matched control by *t test*, with statistical significances indicated (*P<0.05).

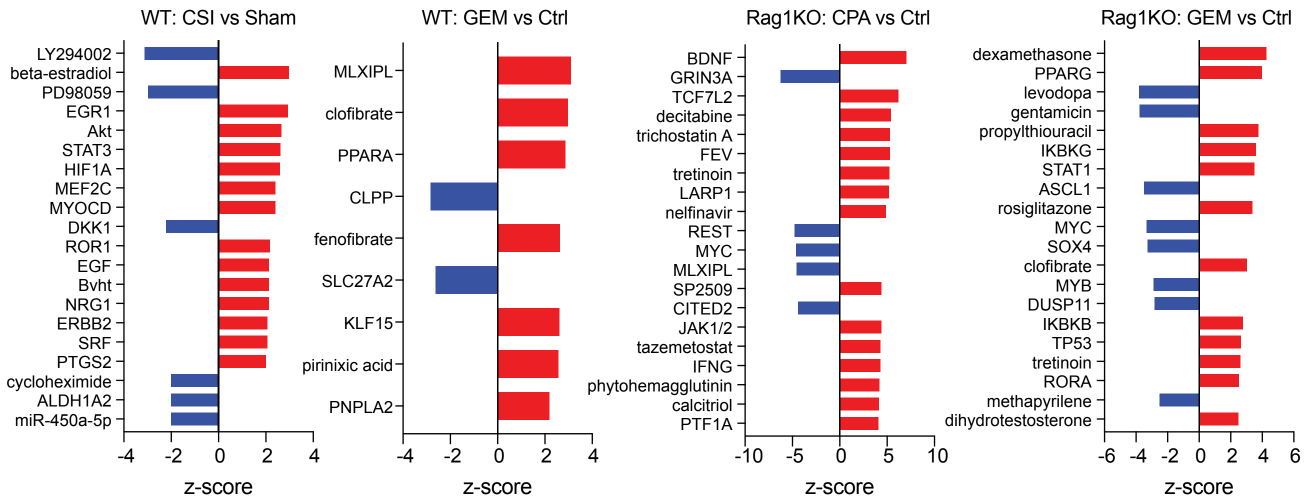

Figure S7. Upstream molecular drivers show limited immune system drivers in mice harboring Myc/p53^{DD} tumors treated with radiotherapy and chemotherapy. RNA sequencing was performed on Myc/p53^{DD} medulloblastomas from either C57Bl/6J WT mice or Rag1KO mice that were harvested upon the development of tumor-related morbidity following treatment with sham, CSI, saline, CPA, or GEM. Differential gene expression analysis is described in the main text. Top 20 upstream molecular drivers determined using KnowledgeBase are shown for each comparison ranked by z-score for contrasts with significantly differentially expressed gene signatures shown (*red=activation, blue=inhibition*). Number of mice in each group were: WT/Sham = 8, WT/CSI = 5, Rag1KO/Sham = 6, Rag1KO/CSI = 5, WT/Ctrl = 4, WT/CPA = 3, WT/GEM = 3, Rag1KO/Ctrl = 4, Rag1KO/CPA = 3, Rag1KO/GEM = 3.

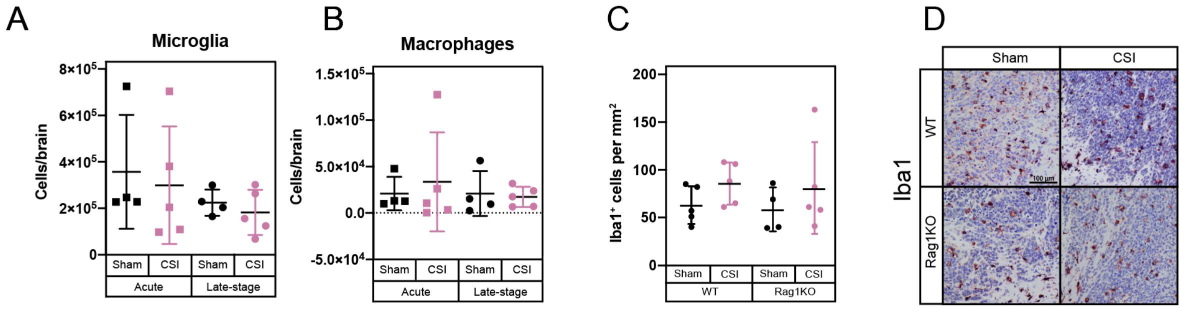

Figure S8. CSI does not induce an increase in macrophages nor microglia in the brain. (A) No differences were observed in absolute counts of microglia or **(B)** macrophages in the brain of CSI treated (*pink*, n=5) WT mice bearing Myc/p53^{DD} tumors compared to control (*black*, n=4), either 24 hours after the final dose (*squares*, labelled "Acute") or by late-stage tumor development (*circles*, labelled "Late-stage"). **(C)** The number of Iba1^{+} cells counted in late-stage Myc/p53^{DD} tumors in C57Bl/6 WT and Rag1KO mice treated with CSI (*pink*, n=5) compared to shams (*black*, n=5) did not differ. **(D)** Representative images of Iba1 staining in late-stage Myc/p53^{DD} tumors in WT and Rag1KO mice treated with sham or CSI. Horizontal bars in graphs indicate mean and error bars indicate standard deviation. Each treatment group was compared to its time-matched controls by *t test.*
